# Improving numeracy through values affirmation enhances decision and STEM outcomes

**DOI:** 10.1371/journal.pone.0180674

**Published:** 2017-07-12

**Authors:** Ellen Peters, Brittany Shoots-Reinhard, Mary Kate Tompkins, Dan Schley, Louise Meilleur, Aleksander Sinayev, Martin Tusler, Laura Wagner, Jennifer Crocker

**Affiliations:** 1 Department of Psychology, The Ohio State University, Columbus, Ohio, United States of America; 2 Department of Marketing, Rotterdam School of Management, Erasmus University, Rotterdam, The Netherlands; Mälardalen University, SWEDEN

## Abstract

Greater numeracy has been correlated with better health and financial outcomes in past studies, but causal effects in adults are unknown. In a 9-week longitudinal study, undergraduate students, all taking a psychology statistics course, were randomly assigned to a control condition or a values-affirmation manipulation intended to improve numeracy. By the final week in the course, the numeracy intervention (statistics-course enrollment combined with values affirmation) enhanced objective numeracy, subjective numeracy, and two decision-related outcomes (financial literacy and health-related behaviors). It also showed positive indirect-only effects on financial outcomes and a series of STEM-related outcomes (course grades, intentions to take more math-intensive courses, later math-intensive courses taken based on academic transcripts). All decision and STEM-related outcome effects were mediated by the changes in objective and/or subjective numeracy and demonstrated similar and robust enhancements. Improvements to abstract numeric reasoning can improve everyday outcomes.

## Introduction

In 2015, only 25% of high school seniors were considered proficient in math [1]. Twenty-nine percent of American adults (about 68 million) are estimated to have only rudimentary math skills, and less than 10% of Americans (about 21 million adults) are thought able to understand complex numeric information [2]. Federal and other programs often target mathematics as one important part of Science Technology Engineering and Math (STEM) education that will lead to better jobs, innovation, improved economy, and greater global leadership. Some educators, however, have questioned whether training beyond basic arithmetic skills is necessary for everyone. Hacker [3], for example, opined “Think of math as a huge boulder we make everyone pull, without assessing what all this pain achieves.” In the present paper, we specifically examined whether improving math skills (numeracy) within a statistics course can justify this pain through its impacts on decision outcomes that people experience.

Improving numeracy is considered important based on its correlations with the quality of decision processes and outcomes [4–6]. Higher numeracy, for example, has been associated with better health outcomes (superior control of stroke risk and diabetes, lower Body Mass Index [7–9]). More numerate individuals also do better financially by having more adequate retirement savings, avoiding predatory loans, and paying credit cards in full [10–12]; numeracy also may be an important underlying factor in financial literacy [13, 14]. Of course, numeracy could be simply an indicator of education or socioeconomic status so that those variables predict better health and financial outcomes rather than numeracy itself. Indeed, prior research [15] has found that education predicts better cognitive skills (including numeracy), higher socioeconomic status, more health-related knowledge, and taking more health-protective behaviors. However, in their study, structural equation modeling indicated that numeracy and other cognitive abilities, and not wealth or health-related knowledge, mediated education’s effects on health-protective behaviors. Furthermore, in a second study conducted in a population from the Peruvian Highlands, results indicated that numeracy was a unique predictor of a health-protective behavior even after accounting for measures of education, other cognitive abilities, and confounding factors [16].

Also unclear is whether these findings are due to objective numeracy (defined as the ability to comprehend and use probabilistic and other mathematical concepts) or subjective numeracy (defined as self-assessed ability and preferences for numbers). Past research often used these constructs interchangeably, and they are positively associated [17]. Objective numeracy, however, has been linked with superior processing and use of numeric information in judgments and decisions independent of general intelligence [5,15], making it a likely cause of decision quality. Independent of objective numeracy, greater subjective numeracy, on the other hand, has been associated with having more motivation, confidence (belief in one’s ability), and positive emotions to numbers, all of which may lead to greater engagement with numbers [18]. Consistent with engaging more with numbers, individuals with higher vs. lower subjective numeracy, but not objective numeracy, were willing to pay more for (highly numeric) genetic screening results [19]. Thus, subjective numeracy may also be important to health and financial outcomes that require persevering with numeric information over time [18]. We tested the potentially separable effects of objective and subjective numeracy.

Here, we report a psychology statistics classroom study that tested the effectiveness of an intervention intended to improve both numeracies and thereby enhance decision outcomes. We also examined the intervention’s effectiveness in improving STEM-related outcomes. Such statistics courses should improve aspects of numeracy including arithmetic and probabilistic reasoning, through practicing related skills on homework and exams. Lehman et al. [20], for example, argued “that psychology and medicine improve statistical and methodological reasoning about a wide range of problems because both fields teach their students how to think about uncertain events, in part through instruction in statistics” (p953, [21]). In the present statistics course, students describe populations and samples using descriptive means, statistical intervals, correlation coefficients, and graphics. They compare and test by detecting differences between statistical populations or reference values using simple hypothesis tests such as t-tests. They predict and explain data using regression. Many of these tests they learn to calculate by hand, and all of this practice should improve their numeracy skills. In fact, Lehman and Nisbett [21] found that different undergraduate training produced different changes in reasoning from the first to the fourth year of university enrollment. Specifically, psychology undergraduates improved considerably in statistical and methodological reasoning but not in conditional reasoning whereas Natural Science and Humanities students demonstrated opposite effects. These undergraduate majors did not differ in changes to nonnumeric verbal reasoning. In related research, taking a statistics course improved one aspect of decision making (use of the law of large numbers [22]).

However, taking math courses, including the present statistics course, involves difficult concepts and negative feedback, which can pressure students considerably (depending on how well student learning is supported), reduce their self-efficacy, and interfere with learning [23, 24]. Thus, the challenge of such courses for improving numeracy is the potential stress of taking the course. This stress can potentially decrease perceived numeric ability and interfere with improvements to objective numeric ability [25]. Indeed, past research has shown that objective numeracy and everyday math anxiety have opposing effects, with greater math anxiety inhibiting confidence and accuracy in calculations and risk interpretations whereas greater objective numeracy improves them [26]. The possibility of stress harming the development of numeracy was particularly likely in the current research because this statistics course: (a) is required for all psychology majors and (b) must be passed with at least a C- before enrolling in major requirements.

A theoretically motivated psychological intervention, called values affirmation [27, 28], in which people reflect on core values, can focus people on long-term goals over immediate pressures [29, 30] and increase acceptance of counter-attitudinal thoughts [31]. As a result, it may increase or protect beliefs in numeric ability and attitudes towards numbers, and ultimately boost performance [28]. According to self-affirmation theory, when people are able to affirm valued aspects of their self-concepts (e.g., by writing about important values), they are reminded of positive aspects of their identities that act as psychological resources for dealing with threats to the self-concept even in unrelated domains [32].

In educational settings like the one studied in the present research, threats to valued self-concepts (e.g., feeling that one is smart or capable) are chronic [33], and they can escalate over the semester (e.g., due to stress [34]), interfering more and more with learning. In particular, students must master early material to learn increasingly difficult concepts, but stress and anxiety about performance can cause poor performance which further increases stress and anxiety and further interferes with learning. Conversely, good performance may improve students’ self-confidence; it can change appraisals about failure so that they are perceived as isolated or temporary events or even as opportunities for improvement which could promote future good performance [32]. As a result, seemingly small manipulations can have oversize effects if administered strategically because of their self-reinforcing and recursive effects [32, 33]. For example, values-affirmation exercises can protect against anxiety and stress about performance [34], prevent poor performance from reducing feelings of academic belongingness [35], reduce rumination about academic failure [36], and buffer against declines in academic self-confidence and perceived academic belongingness [37], all of which ultimately could lead to improved learning, especially in challenging courses. Indeed, lab studies demonstrate that such affirmations can improve performance in difficult topics (e.g., physics [27, 28, 38, 39]), and their effects can persist over multiple school years [27, 28, 39].

In the present study, we used such a values-affirmation manipulation conducted within a college statistics course to experimentally alter numeracy and test its impacts on decision outcomes. Based on prior literature, we expected that a values-affirmation manipulation administered at the beginning of the semester would stave off a recursive cycle of experienced threat from the course and improve development of objective numeracy skills [27, 28, 38, 39]. We also expected improvements to or protection of self-perceptions about ability and attitudes towards numeric information (i.e., subjective numeracy) because of documented protective effects of values-affirmation on perceived ability to succeed at school [37]. Our primary hypothesis concerned the intervention’s possible snowball effects; we expected the increased numeracy to improve the quality of financial literacy, financial outcomes, and health-related behaviors. Such a result would be a generalization of values-affirmation effects not tested previously. Of key interest, it would also support the importance of learning abstract numeric skills and the previously-untested causal relation between numeracy and decision outcomes. In addition, we examined effects on STEM-related outcomes (e.g., statistics course grades, future math-course intentions). Although affirmation can reduce stereotype-threat-induced gender gaps in performance, females must be a minority group for stereotype-threat effects to occur [40, 41]. Because females are in the majority in required psychology courses like the present one, we did not hypothesize stereotype-threat effects.

## Materials and methods

### Ethics statement

This study was conducted in accordance with the ethical standards laid down in the 1964 Declaration of Helsinki. The investigation was approved by the Ohio State University Institutional Review Board (IRB; protocol 2011B0306). Prior to participation, all participants provided written consent by signing an IRB-approved document indicating that they understood the protocol and agreed to participate.

### Participants

Participants were undergraduates recruited from a psychology statistics course that is required for psychology majors and must be passed with at least a C- to enroll in additional psychology courses. A total of 290 students consented and completed at least some measures; 221 students (75% female; ages 17–59, M = 20.9) participated in the intervention and were included in the present paper. Based on a prior meta-analysis [42], we expected small- to medium-sized hypothesized effects. Given expected enrollments and retention, we estimated recruiting from three classrooms would provide adequate power. No effects of classroom existed on hypothesized results, and it is not discussed further.

### Procedure

At the beginning of the term (Time 1), participants completed measures of subjective and objective numeracy, sexist math stereotypes, trait math anxiety, decision-bias and risk-perception tasks (these tasks are unrelated to the present analyses and are not mentioned again), financial outcomes, financial literacy, health-related behaviors, intentions to take more math-intensive classes, science literacy, vocabulary, working memory, and demographics in a two-hour lab session.

Participants were randomly assigned to values-affirmation or control and wrote twice for 10–15 min, once in class (week 2) and again online 2–3 days before the first midterm (week 4). In an online survey at the end of the term (Time 2), participants completed the same measures again (except vocabulary and working memory). The instructors provided final grades. One-two years later, we obtained student transcripts which were coded for the number of math-intensive classes students took before, during, and after participation. We invited participants to complete study materials again after one and two years; however, poor retention resulted in sample sizes too small for appropriate analysis: n_Year1_ = 76 and n_Year2_ = 63 (S1 Supporting Information).

### Independent variable: Values-affirmation manipulation

Using previously successful value-affirmation methods [27–30, 34–39], participants ranked a list of six values (i.e., relationships with family/friends, spiritual/religious values, business/economics, art/music/theater, science/pursuit of knowledge, government/politics) by personal importance. They then wrote about either why their most important value was important and meaningful to them (values-affirmation; *n* = 112) or how their least important value might be important and meaningful to other people (control; *n* = 109). Each group then selected the top two reasons why their chosen value was important to them (or might be important to other people). Thus, both groups wrote about values and their importance, but the exercise was self-relevant only for the intervention group. Course instructors were blind to condition, and students were unaware of the purpose of the writing exercises. Participants responded to the same instructions (values-affirmation or control) each time they completed the manipulation.

### Measures

Participants responded to measures in the order described below. (See S1 Table for descriptive statistics and S2 Table for descriptive statistics by condition).

#### Subjective numeracy scale

(SNS [17]). Participants completed eight items; four items assessing perceived numeric abilities and four items assessing preference for numeric information by responding on 6-point scales from 1–6; higher numbers indicated greater subjective numeracy. Scores were averages across the eight items (range: 1.75–6.00, *M* = 4.22, *SD* = 0.84, α = .81).

#### Sexist math stereotypes

[28]. Participants indicated on 1–5 scales their level of agreement to two statements regarding their stereotypes about gender and math (e.g., “I expect men to generally do better in statistics [math] than women.” Higher numbers indicated higher agreement and sexist math stereotypes. Responses were averaged (range: 1.0–5.0, *M* = 2.60, *SD* = 1.05).

#### Trait math anxiety

[43]. We used a ten-item measure of trait math anxiety (e.g., “I have usually been at ease during math tests”) on a 4-point scale (1 = almost never to 4 = almost always). Responses were reverse-scored as needed and then averaged (range: 1.0–4.0, *M* = 2.50, *SD* = 0.66, α = .90).

#### Objective numeracy scale

(ONS). The ONS measure was comprised of 16 symbolic arithmetic items used successfully to predict decision performance in a prior study [15] (e.g., “126 ÷ 42 = ”, “7/8–2/8 = ”,) and a traditional 18-item numeracy scale assessing skill at probabilistic and other math problems ([44], e.g., “If the chance of getting a disease is 20 out of 100, this would be the same as having a __% chance of getting the disease”). Two traditional numeracy items failed to record properly, leaving 32 items. On the basis of general estimating equations, we summed these two subscales (see results and S1 Supporting Information). The objective numeracy index was the sum of correct answers; higher numbers indicate higher numeracy (range 1–31, *M* = 22.73, *SD* = 5.74, α = .88; see S3 Table).

#### Health-related behaviors

Participants self-reported ten health-related behaviors (e.g., “In the past 3 months, have you had sex in a non-committed relationship without using a condom?”), intentions (e.g., “Do you plan to get a flu vaccine this coming year?”), and habits (e.g., “Do you smoke cigarettes?”). Healthy behaviors were coded as 1, summed, and divided by the total behaviors the participants had the opportunity to experience (range: 0.2–1, *M* = 0.64, *SD* = 0.18, higher scores indicate better behaviors; see S1 Supporting Information and S4 Table for the wording of all items and their results at the two timepoints).

#### Financial outcomes

Participants self-reported ten yes-or-no questions assessing financial behaviors and knowledge (e.g., “Do you have a savings account or emergency fund?”, “Do you know your credit card balance?”). Positive financial outcomes were coded as 1, summed, and divided by the total number of outcomes the participants had the opportunity to experience, with higher numbers indicating better outcomes (range: 0–1, *M* = 0.79, *SD* = 0.21; see S1 Supporting Information and S4 Table).

#### Intentions to take additional math classes

Students were asked “How likely are you to take another class that involves a lot of math?” on a scale from 0 (not at all likely) to 6 (very likely) (range: 0–6 *M* = 2.27, *SD* = 2.06).

#### Financial literacy

Participants answered 5 investment-related questions [11] (e.g., “considering a long time period (e.g., 10 or 20 years), which asset normally gives the highest return?”). One ambiguous item was removed from analysis (see S1 Supporting Information). Correct answers were summed; higher numbers indicate greater literacy (range: 0–4, *M* = 1.88, *SD* = 1.16; see S1 Supporting Information and S4 Table).

#### Science literacy

[45]. Participants answered 8 true/false science-literacy questions (range: 2–8, *M* = 6.68, *SD* = 1.32).

#### Vocabulary

[46]. Participants were tested on the meaning of 36 target words (range: 1–30, *M* = 19.54, *SD* = 5.32, α = .82).

#### Working memory

[47]. Working memory was assessed via a backwards letter sequence task (range: 2–9, *M* = 5.17, *SD* = 1.29).

#### Final grades

Final grades (out of 100) included homework assignments and three exams. Extra credit was not included in analyses (range: 17.40–97.91, *M* = 80.03, *SD* = 13.21).

#### Math-intensive course enrollment

We obtained full transcripts for consenting participants approximately two years later and coded for math-intensive classes. For each participant, the number of math courses taken was divided by the number of terms, separately for pre- and post-experiment. Retaking the current statistics class was not counted as an additional math-intensive class. Courses taken during the experimental term were counted as pre-experiment. The average number of math-intensive post-experiment courses taken ranged from 0 to 1.6 (*M* = 0.14, *SD* = 0.27).

## Results

Retention analyses using logistic regression revealed no significant interactions with intervention condition for Time-1 numeracy measures, demographics, academic term, or instructor (S1 Supporting Information). In the final dataset, no condition differences existed for general intelligence (vocabulary, working memory) or other predictors. However, a greater proportion of Whites existed in intervention than control (χ^2^ (1) = 7.64, *p* = .007; S2 Table) and intervention participants reported worse Time-1 financial outcomes than control in a repeated-measures analysis of variance (RMANOVA) (*M*_*InterventionFinOuttime1*_ = 0.78, *M*_*ControlFinOuttime1*_ = 0.83, *F*_1,191_ = 4.01, *p* = .047, η^2^ = .021). No other demographic or outcome differences existed between conditions at Time 1. We controlled for ethnicity and Time-1 financial outcomes in all analyses. Excluding them did not substantially alter effects (S5 Table for correlations between all variables included in analyses).

In the analyses that follow, we generally used RMANOVAs to examine total effects of the intervention from the beginning to the end of the academic term. The one exception was objective numeracy; for it, we used generalized estimating equation (GEE) analysis that allowed us to test whether the intervention differentially affected its two subscales across time, a three-way interaction with two repeated factors. For decision and STEM-related outcomes, we then examined structural equation models (SEMs) to examine possible mediating effects of numeracy.

### Subjective and objective numeracy: Tests of total effects

**Fig 1:**
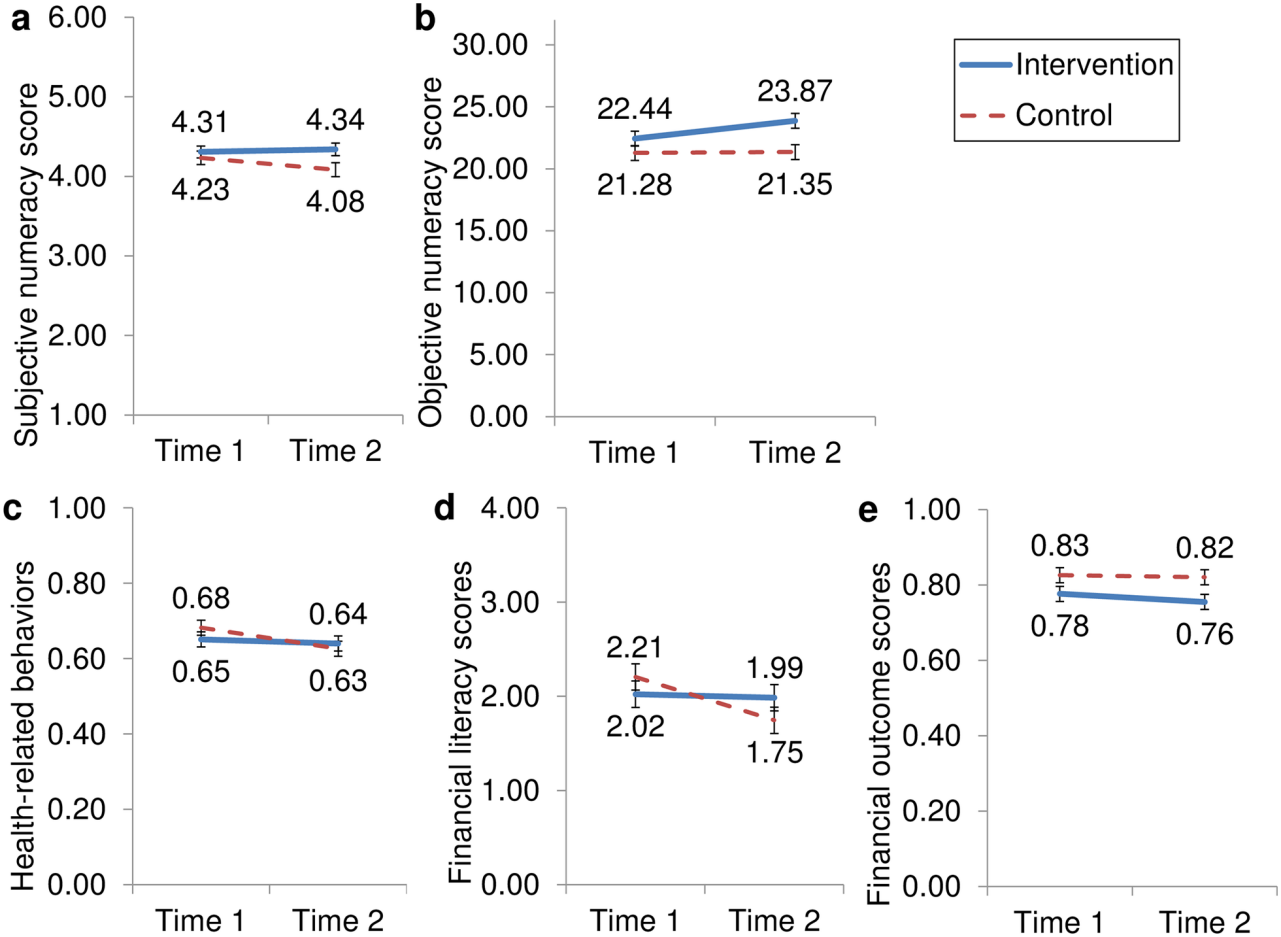
Total effects of intervention on numeracy and decision outcomes. Estimated marginal means of intervention (vs. control) from RMANOVAs on (A) subjective numeracy (SNS), range = 1–6, *n* = 199; (B) objective numeracy (ONS), out of 32 possible, *n* = 187; (C) health-related behaviors, range 0–1, *n* = 194; (D) financial literacy, range = 0–5, *n* = 193; and (E) financial outcomes, range 0–1, *n* = 194; all measured at the beginning (Time 1) and end (Time 2) of the academic term, with ethnicity and Time-1 financial outcomes entered as covariates. In each case, higher numbers means better performance or outcomes. Error bars indicate ± 1 *SE*.

As hypothesized, the intervention had positive effects on both objective-numeracy and subjective-numeracy scores. In an RMANOVA of subjective numeracy (*n* = 194, see Fig 1A), the intervention protected subjective numeracy over the term (interaction *F*_1,190_ = 3.96, *p* = .048, η^2^ = .020), with SNS scores declining in the control condition (*M*_*SNStime1*_ = 4.23, *M*_*SNStime2*_ = 4.08; *F*_1,190_ = 5.31, *p* = .022, η^2^ = .027) and remaining stable in the intervention condition (*M*_*SNStime1*_ = 4.31, *M*_*SNStime2*_ = 4.34; *F*<1). The intervention’s effect was significant at Time 2, *F*_1,190_ = 4.25, *p* = .041, η^2^ = .022. No effects of the covariates emerged.

We conducted a GEE analysis of objective numeracy with ONS divided into its two subscales—traditional numeracy and symbolic arithmetic—to test whether values-affirmation had differential effects. Subscale and time were both entered as within-participant variables and ethnicity and Time-1 financial outcomes were entered as covariates (the covariates did not significantly predict ONS scores). We used maximum likelihood estimation with a normal probability distribution, identity link function, and independent correlation matrix to examine the three-way interaction of ONS subscale (symbolic arithmetic vs. traditional numeracy) × time (Time 1 vs. Time 2) × values-affirmation condition (values affirmation vs. control).

The three-way interaction was nonsignificant (*p* = .232), indicating that the affirmation intervention did not have differential effects on the two subscales. We then removed nonsignificant factors one at a time, beginning with the highest order interaction, and reran the model each time. The final model included a significant main effect of time, such that ONS was higher at Time 2, Wald χ^2^(1) = 4.21, *p* = .040, and a main effect of affirmation, such that scores were higher for participants who affirmed their values, Wald χ^2^(1) = 7.04, *p* = .008. A significant interaction also emerged of time and ONS subscale, Wald χ^2^(1) = 20.10, *p* < .001, such that, collapsed across affirmation condition, performance on symbolic arithmetic problems improved over the semester (M_time 1_ = 10.75, M_time 2_ = 11.73), Wald χ^2^(1) = 12.30, *p* < .001, whereas performance on the traditional numeracy subscale was similar from Time 1 to Time 2 (M_time 1_ = 11.06, M_time 2_ = 10.91, Wald χ^2^<1). The difference between the subscales was significant at Time 2, Wald χ^2^(1) = 11.89, *p* = .001.

Importantly and as hypothesized, ONS (as represented by both subscales) improved over the semester for the intervention group (M_time 1_ = 11.14, M_time 2_ = 11.92, Wald χ^2^(1) = 11.90, *p* = .001), but it did not improve among control participants (M_time 1_ = 10.66, M_time 2_ = 10.71, Wald χ^2^<1; time × affirmation interaction: Wald χ^2^(1) = 3.24, *p* = .072; Fig 1b). The effect of condition was significant at Time 2, Wald χ^2^(1) = 8.58, *p* < .001. We retested this two-way interaction using RMANOVA of the summed objective-numeracy index. Results were substantially similar, but the interaction of time and condition did not reach significance (*p* = .116; see S1 Supporting information and Fig 1B).

Results of two additional RMANOVAs revealed that the intervention did not affect performance on a non-numeracy-related test of scientific literacy (*n* = 193, *F*<1) or self-reported trait math anxiety (*n* = 194, *F*<1).

### Decision outcomes: Tests of total effects (RMANOVAs) and numeracy-mediated indirect effects (SEMs)

Conducting RMANOVAs of each decision outcome revealed supportive evidence for two of three hypothesized causal effects. First, the intervention marginally protected health-related behaviors (*n* = 194) from the beginning to end of the term (Fig 1C, interaction *F*_1,190_ = 3.73, *p* = .055, η^2^ = .019). Students in the control condition declined significantly in reported health-related behaviors by Time 2 (*M*_*Healthtime1*_ = 0.68, *M*_*Healthtime2*_ = 0.63; *F*_1,190_ = 11.53, *p* = .001, η^2^ = .057) whereas health-related behaviors were protected in the intervention condition (*M*_*Healthtime1*_ = 0.65, *M*_*Healthtime2*_ = 0.64; *F*<1). Overall, health-related behaviors declined from Time 1 to Time 2, *F*_1,190_ = 4.05, p = .045, η^2^ = .021; no effects of Time-1 financial outcomes or ethnicity emerged. The intervention also protected financial literacy (Fig 1D, *n* = 193, interaction *F*_1,189_ = 6.25, *p* = 0.013, η^2^ = .032), with no change for those in the intervention (*M*_*FinLittime1*_ = 2.02, *M*_*FinLittime2*_ = 1.99; *F*<1) while control participants declined in financial literacy (*M*_*FinLittime1*_ = 2.21, *M*_*FinLittime2*_ = 1.75; *F*_1,189_ = 14.58, *p* < .001, η^2^ = .072); no effects of Time-1 financial outcomes or ethnicity emerged. For financial outcomes, no significant total effect of the intervention emerged (Fig 1E, *F* = 1, *n* = 194). However, the effect of random assignment to condition on Time-1 financial outcomes persisted into Time 2 (*F*_1,191_ = 6.76, *p* = .01, η^2^ = .034) so that financial outcomes were worse for intervention participants than control (S2 Table). Effects of time and ethnicity on financial outcomes also existed; financial outcomes declined over the academic term (*F*_1,191_ = 6.02, *p* = .015, η^2^ = .031), and white participants reported better outcomes (F_1,191_ = 6.23, *p* = .013, η^2^ = .032). The effect of ethnicity on financial outcomes was stronger at Time 2 (interaction F_1,191_ = 3.88, *p* = .050, η^2^ = .020. For item-by-item analysis of these outcomes by intervention condition and time, see S1 Supporting Information and S6 Table)

**Fig 2:**
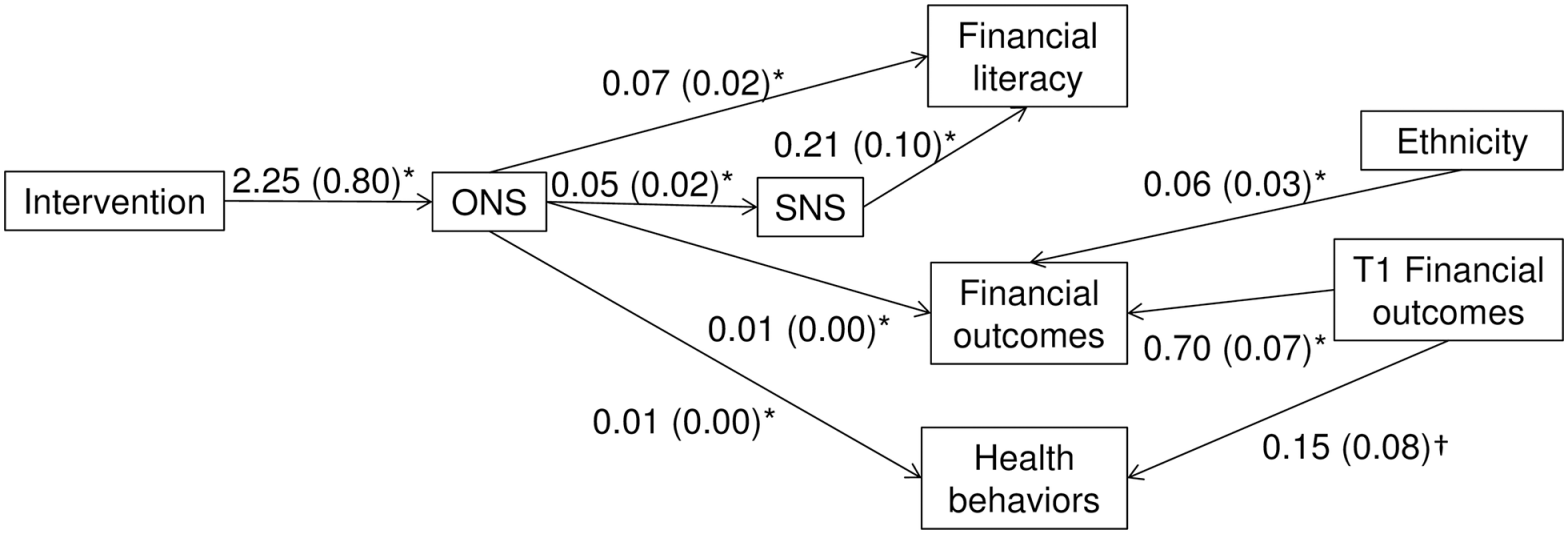
Numeracy-mediated indirect effects on decision outcomes. Structural equation model of the effects of the numeracy intervention on financial literacy, financial outcomes, and health-related behaviors (*n* = 194), all assessed at the end of the term. Paths with *p>*.100 were removed from the model; *p<0.05, †p < .10. An unexpected negative indirect-only effect emerged of the intervention on Financial outcomes (not shown; B = -0.06, *SE* = 0.02). Path coefficients are unstandardized; standard errors are in parentheses. ONS = objective numeracy; SNS = subjective numeracy.

To examine hypothesized effects of improved numeracy on Time-2 decision outcomes, we conducted an SEM of the three decision outcomes with the intervention leading to objective numeracy and then subjective numeracy (all measured at Time 2 and with numeracy measures used as serial mediators) and with all possible paths to each outcome. Because affirmation could have global effects independent of numeracy or numeracy-mediated effects on our outcomes (e.g., a general effect of affirmation to reduce stress independent of numeracy), we included paths from affirmation to each outcome variable. We also allowed for direct effects of objective numeracy on outcomes in addition to the indirect effects through subjective numeracy, because we were interested in whether objective numeracy or subjective numeracy would matter most. We also included paths for Time-1 financial outcomes and ethnicity on ONS, SNS, and each outcome because both variables were related to condition at Time 1. We then eliminated nonsignificant paths one at a time (see S1 Fig for starting model). We used Mplus software and maximum likelihood estimation with conventional standard errors and chi-square test statistic using all available data. Indirect (mediated) effects of the intervention on final outcomes were estimated using bootstrapping (5,000 samples). Although the model allowed outcomes to covary, no significant covariation existed (*p*s>.410). A similar model in which the intervention improved objective numeracy through subjective numeracy did not fit the data as well as indicated by Bayesian Information Criteria (BIC [48]; S2 Fig). The final SEM fit the data well (see Fig 2; n = 194; χ^2^(12) = 6.16, *p* = .908; RMSEA = 0.00 [90%CI: 0.00 to 0.03]; CFI = 1.00; AIC = 1,978.34; BIC = 2,053.50).

First and as hypothesized, the superior Time-2 health-related behaviors produced by the intervention were mediated by numeracy. Improvements to objective but not subjective numeracy mattered. The intervention produced greater objective numeracy which led to more healthy behaviors (indirect effect (IE) = 0.02, *SE* = 0.01, 95%CI: 0.002, 0.03). An effect of Time-1 financial outcomes also emerged such that better Time-1 financial outcomes predicted better Time-2 health-related behaviors.

Second, the intervention’s total effect on financial literacy was mediated by numeracy. Improvements to both objective and subjective numeracy improved financial literacy (total IE = 0.17, *SE* = .06 95%CI: 0.05, 0.30). The intervention’s IE through objective numeracy was 0.15 (*SE* = 0.06, 95%CI: 0.04, 0.27) and through both objective and subjective numeracy was 0.02 (*SE* = 0.02, 95%CI: 0.001, 0.07).

Finally, for financial outcomes, although an intervention-related total effect was not detected, the intervention had a positive indirect-only effect on financial outcomes through objective numeracy (IE = 0.02, *SE* = 0.01, 95%CI: 0.002, 0.03). In addition, Time-1 financial outcomes predicted Time-2 financial outcomes, and an effect of ethnicity was found (nonwhite participants had worse outcomes than white participants). The intervention also had an unexpected negative indirect-only effect on Time-2 financial outcomes after controlling for covariates and the intervention’s numeracy-mediated positive effect.

### STEM-related outcomes: Tests of total effects and numeracy-mediated indirect effects (SEMs)

Conducting RMANOVAs for math-course intentions (*n* = 192) and average math-intensive courses per term (*n* = 179) revealed no significant interactions for condition over time (*F*s<1). Participants did take more math-intensive courses, on average, pre- than post-experiment, *F*_1,175_ = 17.74, *p* < .001, η^2^ = .092, and nonwhite participants took more math-intensive courses than white participants, *F*_1,175_ = 5.71, *p* = .018, η^2^ = .032. Based on ANOVA, the intervention did not improve course grades (*n* = 206, *F*<1; S2 Table). No other significant effects emerged.

To examine hypothesized effects of improved numeracy on Time-2 STEM-related outcomes, we conducted similar SEM models as before by modeling possible effects of the intervention on STEM-related outcomes (through objective and then subjective numeracy as serial mediators). We included paths from Time-1 financial outcomes and ethnicity to ONS, SNS, and each outcome variable (see S1 Supporting Information and S3 Fig for starting model). A similar model in which the intervention affected subjective numeracy first and then objective numeracy did not fit the data as well ([46] BIC difference = 15.07, S1 Supporting Information and S4 Fig).

**Fig 3:**
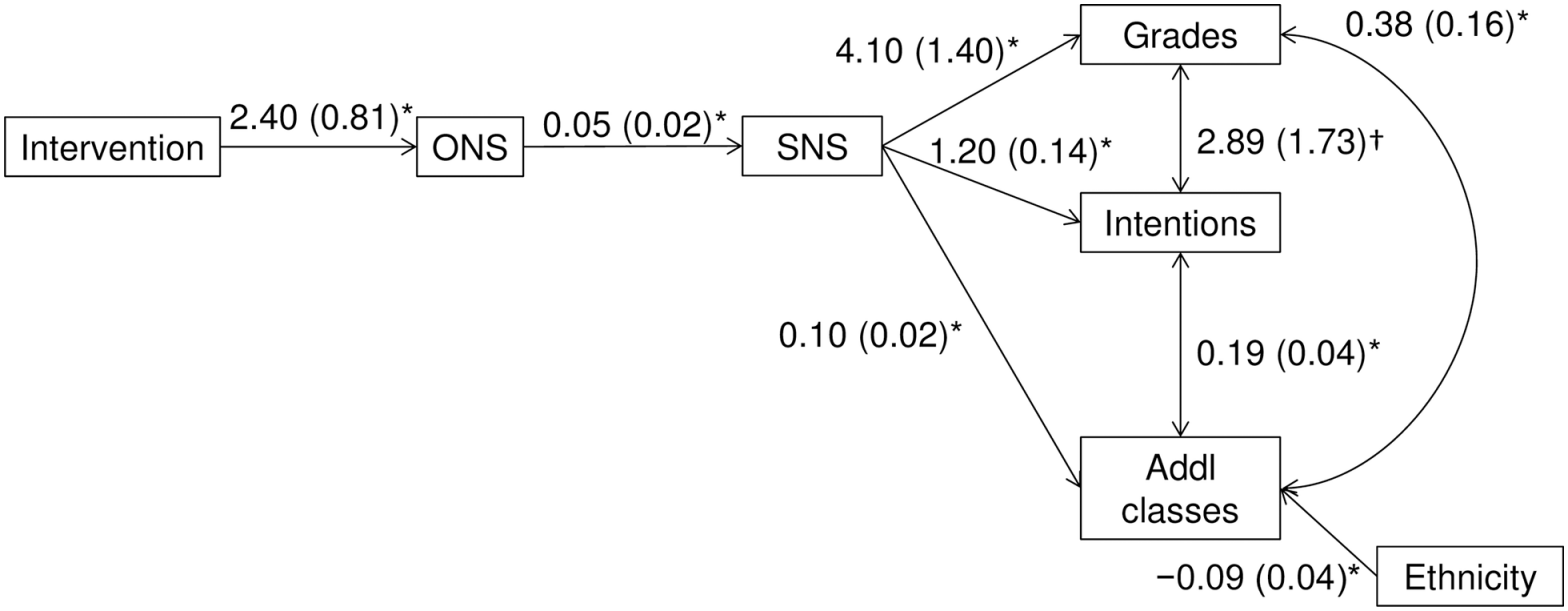
Numeracy-mediated indirect effects on STEM-related outcomes Structural equation model of the effects of the numeracy intervention on grades in the statistics course, future intentions to take more math-related courses, and additional math-intensive courses taken per term (*n* = 218). Paths with *p>*.100 were removed from the model; the remaining paths are *p < .050, †p < .10. Path coefficients are unstandardized; standard errors are in parentheses. ONS = objective numeracy; SNS = subjective numeracy.

Although the intervention did not demonstrate the hypothesized causal effects on STEM-related outcomes, the intervention had a positive effect, as expected, through objective and subjective numeracy to provide similar patterns of improvements for all three STEM-related outcomes (see Fig 3). The final SEM fit the data well (*n* = 218; χ^2^(11) = 11.54, *p* = .399; RMSEA = 0.02 [90%CI: 0.00 to 0.07]; CFI = 1.00; AIC = 4,196.42; BIC = 4,260.72). The intervention produced greater Time-2 ONS which led to greater SNS and superior STEM-related outcomes in turn (grades: IE = 0.53, *SE* = 0.31, 95%CI: 0.09, 1.25; for intentions: IE = 0.16, *SE* = 0.07, 95%CI: 0.04, 0.32; additional post-experiment math-intensive courses: IE = 0.01, *SE* = 0.01, 95%CI: 0.003, 0.03). Nonwhites took more math-intensive courses. The STEM-related outcomes covaried with one another (see Fig 3). Time-1 financial outcomes were removed from the final model for nonsignificance. For each STEM-related outcome, having greater SNS was the most proximal factor in improvements.

### Did affirmation affect numeric efficacy, attitudes, or both?

Past research suggests that motivating behaviors, such as those in the present study, requires multiple changes: in perceptions of the ease or difficulty of performing the behavior (efficacy), attitudes towards the behavior, and subjective norms [49]. The subjective-numeracy scale has subscales that represent the first two of these components (perceived ability and number preferences, respectively), allowing us to explore how far affirmation’s effects extended over the time course of the present study. The affirmation literature suggests positive effects might exist for both subscales due to its demonstrated effects on self-efficacy [37] and attitudes [31]. In RMANOVAs similar to those conducted for the full SNS scale, the intervention had a positive effect on perceived-ability scores, but no significant effect on number preferences. For SNS-ability (*n* = 194), the intervention marginally protected perceived ability (interaction *F*_1,190_ = 3.14, *p* = .078, η^2^ = .016), with scores declining in the control condition (*M*_SNS-Abilitytime1_ = 4.03, *M*_SNS-Abilitytime2_ = 3.81; *F*_1,190_ = 7.31, *p* = .007, η^2^ = .037), and remaining stable in the intervention condition (*M*_SNS-Abilitytime1_ = 4.20, *M*_SNS-Abilitytime2_ = 4.18; *F*<1). The intervention’s effect was significant at Time 2, *F*_1,190_ = 4.97, *p* = .027, η^2^ = .025. No other significant predictors emerged except average perceived ability was marginally higher in the intervention condition (*F*_1,190_ = 3.34, *p* = .069, η^2^ = .017). A second RMANOVA of number preferences (*n* = 194) revealed no significant intervention effects (*M*_SNS-Preferencetime1_ = 4.41, *M*_SNS-Preferencetime2_ = 4.50; interaction *F*_1,190_ = 1.92, *p* = .167) vs. control (*M*_SNS-Preferencetime1_ = 4.44, *M*_SNS-Preferencetime2_ = 4.36). Thus, values affirmation affected perceived efficacy with numbers, but not attitudes towards numbers, over this time period.

## Discussion

In this paper, we present the first known evidence to support numeracy’s causal effects on financial literacy and health-related behaviors. Supporting learning in a statistics course through an affirmation intervention produced positive, albeit small, differences over time for subjective and objective numeracy and generalized to the seemingly unrelated domains of financial literacy and health-related behaviors. The results illustrate how a social psychological approach can be leveraged to improve abstract numeric reasoning and important societal problems in turn. The results held eight weeks after the initial intervention. A further snowball effect may encourage longer term improvements in health and finances [50].

The generalizability of the intervention’s effects to financial literacy and health-related behaviors occurred despite abstract mathematical knowledge being difficult to acquire and especially difficult to transfer successfully to novel situations [51, 52]. The values-affirmation intervention was intended to alter students’ perceptions of the statistics-class setting as one in which their values were taken seriously and therefore in which they could succeed. However, its effects appeared to generalize considerably beyond the intervention’s setting, first, to the present study’s mediators, objective and subjective numeracy, which were closely related to the course content, and, second, to financial literacy and health-related behaviors, which have been correlated with numeracy in past studies. Science literacy, also measured with a test but unrelated to numeracy, was unaffected by the intervention. These comparisons suggest greater generalization of the intervention to related than unrelated contexts. At the same time, the differential intervention effects on perceived efficacy vs. number preferences suggest that the intervention may affect performance efficacy while leaving unaffected how much participants like the topic; related research leaves open the possibility that number preferences too may change with more experience and time [53].

The SEM analyses further revealed positive and robust effects of improving numeracy across qualitatively different decision and STEM-related outcomes (e.g., an objective test, future courses taken, and self-reported outcomes). For STEM-related and financial outcomes, however, the lack of total effects from the intervention leads us to correlational conclusions: Greater numeracy was associated with superior STEM-related and financial outcomes and may encourage individuals to take other math or statistics courses, to start careers in STEM disciplines, and to achieve better financial outcomes. The intervention’s causal effect on financial literacy further supports this latter statement. Overall, the robust SEM results suggest that a continued search for causal effects with financial and STEM-related outcomes may ultimately prove fruitful.

The better-fitting SEM models also indicated that the intervention improved subjective numeracy through its improvements to objective numeracy. Given past research on the relation of values affirmation to stress reduction [34, 54], we might have expected the opposite (although similar results to the present ones emerged in Peters & Bjalkebring [18]). However, these data do not preclude an interactive model in which values affirmation initially reduced the stress of taking the course and improved subjective numeracy earlier (when we did not measure it) and then these improvements led to further reciprocity between objective and subjective numeracy later in the term.

The results also were consistent with the notion that both objective and subjective numeracy matter to decision outcomes, but operate through different psychological processes [18]. Objective numeracy was the proximal cause of the decision-outcome effects eight weeks after the initial manipulation (with subjective numeracy also mattering to financial literacy); subjective numeracy was the more proximal cause of improvements to STEM-related outcomes. It may be that mathematical operations and risk recognition (linked with objective numeracy) are most critical to health-related behaviors and financial outcomes whereas motivation, effort, and confidence with numbers (linked with subjective numeracy) are more critical to STEM-related outcomes [18].

For health-related behaviors, for example, we know from past literature that more objectively numerate individuals tend to take on more health-protective behaviors than the less numerate [55]. This link may be due to the highly numerate using relevant objective probabilities more in their choices [5, 15] whereas the less objectively numerate rely on other heuristic processes such as the use of information frames, mood states, narrative information, and other irrelevant incidental sources of affect (e.g., [55, 56). It may be that intervention participants in the present study were more likely to consider, for example, the statistical odds of pregnancy and ill effects from not exercising when making decisions about sex and exercise-related health-protective behaviors. Control participants, who did not improve in objective numeracy, may have relied more instead on the joy of sex and avoidance of the pain of exercise, for example, to make their decisions. Because many of the health-related behaviors are common and changeable in a short time period, it is certainly possible that health-behavior changes could have emerged during this time period if numeracy began to improve early enough among the self-affirmed and/or to decline early enough in the control condition. We do not have direct evidence for these conjectures, however, and more research is needed.

We also do not have a definitive explanation for why we primarily showed protective effects on outcomes. In general, “self-affirmation theory proposes that people are motivated to protect their view of themselves as good, moral, and efficacious” [33, p279], suggesting a protective effect for subjective numeracy in particular. Such protective effects are not uncommon in this literature. For example, values-affirmation interventions have prevented declines in academic performance [39] and perceived academic potential and belongingness [37]. It may be that college and other students, immediately prior to finals week (the present study’s Time 2), were worn down, stressed, and less motivated, leading them to question their abilities more, perform less well, and choose healthy behaviors less often. The intervention may have reduced such effects as in past research [34, 54]. For financial literacy items, our overall effect was protective, but intervention participants did improve on two items. Although we cannot be sure of the explanation without further studies, we believe that affirmation reduced defensiveness to the numeracy-related material in these items and better allowed participants to make calculations, retrieve number-related knowledge, conduct numeric comparisons, and use logical inferences with number-related materials.

Although we believe that these protective effects were due to values-affirmation preventing declines that ordinarily take place over the course of the academic term and immediately prior to finals week), one could also reasonably attribute control-participant declines to reduced conscientiousness, general stress reactions, or some other unidentified more global response to the values-affirmation manipulation [57] in completing measures at Time 2. If that were the case, one would expect worse performance and evidence of less effort from the control group across all measures rather than only for numeracy-related measures. If the effects were due instead to values-affirmation in a statistics course as a numeracy intervention, however, we would expect that control participants would put less effort than intervention participants into numeracy-related tasks, but similar effort into non-numeracy-related tasks.

We tested these alternative explanations in three ways (see S1 Supporting Information). First, the values-affirmation manipulation did not influence performance on science literacy, a measure unrelated to numeracy. Second and at Time 2, we found that control participants spent less time on the symbolic-arithmetic portion of the ONS measure than did intervention participants (response times were not measured for the traditional numeracy items), but no difference existed between groups for time spent answering the science literacy questions. Finally, we examined the number of words participants wrote in response to an open-ended prompt about the purpose of the study at the end of the academic term (Time 2). If control participants were less conscientious overall, it seems likely that they would have written fewer words or skipped the question altogether. Instead, we found that control and intervention participants wrote the same number of words on average. Taken together, these results across several different possible measures of conscientiousness suggest that values-affirmation vs. control was associated with greater motivation on numeracy-related tasks; the data did not support control participants simply putting less effort into all tasks.

The intervention’s effects instead could have been due to practice effects (e.g., for objective numeracy), but ONS scores improved in the intervention condition with a null effect in the control condition. The lack of intervention effects on science-literacy scores, which also could have shown practice effects, further softened possible practice-effect concerns.

The intervention did have an unexpected negative partial effect on Time-2 financial outcomes after controlling for the intervention’s positive numeracy-mediated effect and Time-1 financial outcomes; this result may indicate a missing mediator [58]. For example, affirming one’s values may have improved financial outcomes through its positive numeracy effects while simultaneously reducing perceived financial stress, making these college students less careful than they might otherwise be about their finances.

The inconsistency between the results for financial outcomes and financial literacy may be explained by the fact that they measure different constructs. Financial outcomes do include knowledge, but it is knowledge about one’s own financial situation, whereas the financial literacy items tested knowledge of general financial principles (as well as calculations and comparisons between numbers). It may be that greater financial literacy ultimately will lead (at a later time) to better financial outcomes among these student participants.

The intervention produced relatively small effects for financial literacy and health-related behaviors and no total effects for financial or STEM outcomes; it would be helpful to understand how to increase these effect sizes. One possibility is through better measurement. For health-related behaviors and financial outcomes, in particular, insufficient time may have passed to detect much change (e.g., in starting up a savings or emergency fund). In addition, some items were predicated by “in the past year”, so that reported negative outcomes may have preceded the manipulation (we had planned to assess intervention effects one and two years after the statistics course, but were unable to recruit sufficient participants; see S1 Supporting Information). Second, we may be able to increase the manipulation’s strength. Our exploratory SNS analyses revealed that the intervention improved perceived ability, but not attitudes towards using numbers and perhaps not subjective norms [49]. Future research should examine whether a values-affirmation manipulation has stronger effects when used in combination with an attitude and/or norm intervention. Alternatively, combining a values-affirmation manipulation with a numeracy intervention more focused than taking a required statistics course may improve effects.

The present study did not replicate prior results demonstrating that females who held negative gender stereotypes about science or math experienced better outcomes following values affirmation [28, 38, S1 Supporting Information]. However, the present results and 75% female sample are consistent with past research showing that classrooms comprised mostly of women minimize stereotype-threat effects [40, 41]. The intervention’s effects also were not stronger for individuals who had higher Time-1 trait math anxiety (S1 Supporting Information) although this finding was limited by our measure which focused on past reactions rather than future expectations. The math anxiety measure we chose may have been too focused on past reactions (e.g., “I have usually been at ease during math tests”). In hindsight, we should have chosen a measure focused on current feelings and their projections onto future expectations (e.g., the everyday math anxiety scale, [26])

In addition, we cannot rule out the possibility that the present effects are limited in their generalizability because of our use of undergraduate psychology students. Although our psychology students are not known for having high math skills and interest in taking this required statistics course, more educated samples are more numerate than the population at large [14]. Values-affirmation effects, however, are usually stronger among vulnerable populations (e.g., first-generation college students [59], women [28, 38], or minorities [27, 35] and have been found in secondary educational settings [27, 35] and with poor community members [60]. Given that the sample was predominately female, our results also may be particularly applicable to female populations. Because women tend to have lower financial literacy [61] and lower numeracy (e.g., [44]) than men, our results suggest that future research should examine whether numeracy interventions could reduce the gender gap in financial literacy.

The results do replicate prior results that math education can fail to produce desired outcomes unless conducted in a supportive environment [23, 24]. In the current sample, only affirmed students showed the expected benefits of the statistics course. In contrast, the control group’s objective numeracy remained unchanged over the academic term, and they became less confident in their numerical abilities.

Taking math and statistics courses has long been considered important for STEM-related careers and national outcomes. Less attention has been paid, however, to the ubiquity of numerical information in health and financial decisions (e.g., mortgages, credit card debt, medical-treatment options). Based on the present data, improving math skills will also directly and/or indirectly improve decision outcomes in these areas. When adequate support is provided for learning [24], it appears worth the pain of pulling that “huge boulder” [3].

## Supporting information

S1 Supporting information Supporting information. Additional procedural detail and analyses.
(DOCX)

S1 Table Descriptive statistics for measures. Descriptives for all measures discussed in the main text by time.
(DOCX)

S2 Table Measures and demographics by time and experimental condition. Descriptive statistics (frequency and percentage or mean and standard deviation) for all measures and demographics for each condition at each time.
(DOCX)

S3 Table ONS items with scoring criteria and percentage correct. (DOCX)

S4 Table Health-related behavior, financial outcome, and financial literacy measures. Percentages of positive responses to health-related behaviors, financial outcomes and financial literacy measures.
(DOCX)

S5 Table Correlation table. Correlation table of all variables included in analyses, restricted to those who participated in the intervention manipulation.
(DOCX)

S6 Table Health-related behavior, financial outcome, and financial literacy measures by condition and time. Percentages of responses to health-related behavior, financial outcome, and financial literacy measures by condition and time.
(DOCX)

S7 Table Beta coefficients for starting SEM models. Beta coefficients (standard error in parentheses) of the effects of covariates (i.e., ethnicity and Time-1 Financial Outcomes) on variables in full SEM model (see S1 Supporting Information, S1 and S3 Figs).
(DOCX)

S1 Fig Starting SEM model for decision outcomes. Structural equation model of the effects of the numeracy intervention on all Time-2 decision outcomes (i.e., financial literacy, health-related behaviors, financial outcomes, n = 194). Path coefficients are unstandardized. Solid paths are p < .0.10, *p < .05, †p < .10, dashed paths are p>.10. Paths from Time-1 financial outcomes and ethnicity are not shown in figure and can be found in S7 Table. ONS = objective numeracy; SNS = subjective numeracy.
(TIF)

S2 Fig Alternative SEM for decision outcomes. Structural equation model of the effects of the numeracy intervention on all Time-2 decision outcomes (i.e., financial literacy, health-related behaviors, financial outcomes, *n* = 194). Path coefficients are unstandardized; standard errors are in parentheses. Paths are *p < .050, †*p* < .010. Nonsignificant paths (*p>*.100) were removed from the final model and are not shown. ONS = objective numeracy; SNS = subjective numeracy.
(TIF)

S3 Fig Starting SEM model for STEM outcomes. Structural equation model of the effects of the numeracy intervention on all Time-2 STEM-related outcomes (i.e., grades in the statistics course, Time-2 intentions to take more math classes, and number of math-intensive courses per term after the experiment, n = 212). Path coefficients are unstandardized; standard errors are in parentheses. Solid paths are *p < .05, †p < .10, dashed paths are p>.10. Paths from Time-1 financial outcomes and ethnicity are not shown in figure and can be found in S7 Table. ONS = objective numeracy; SNS = subjective numeracy.
(TIF)

S4 Fig Alternative SEM for STEM outcomes. Structural equation model of the effects of the numeracy intervention on all Time-2 STEM-related outcomes (i.e., grades in the statistics course, Time-2 intentions to take more math classes, and number of math-intensive courses per term after the experiment, n = 218). Path coefficients are unstandardized; standard errors are in parentheses. Paths are *p < .050, †*p* < .010. Nonsignificant paths (*p*>.100) were removed from the final model and are not shown. ONS = objective numeracy; SNS = subjective numeracy.
(TIF)

S1 File Analyzed data file. (XLSX)
