# Supplementary material for: Improving numeracy through values affirmation enhances decision and STEM outcomes
Source: PLoS One. 2017 Jul 12;12(7):e0180674. doi: 10.1371/journal.pone.0180674 (PMC5507517; doi:10.1371/journal.pone.0180674)
Supplement: S1 Supporting information — Additional procedural detail and analyses. (DOCX) [file pone.0180674.s001.docx]

**S1. Supporting information.**

Additional procedure detail

*Retention efforts for Years 1 and 2*. Participants received up to 5% extra credit in their course for participating in the study. To encourage retention, they were also offered a chance to win one of five $100 cash prizes for completing the last data collection point at the end of the term. Students were given the option of completing paper assignments to earn the same amount of extra credit or course credit as an alternative to participating in the study. For study completion at Year 1 and Year 2, participants were offered, respectively, a $15 and $20 Amazon gift card. Participants were contacted up to six separate times at Years 1 and 2 to encourage retention.

*Missing data in health-related behavior and financial outcome items*. If participants did not respond to an outcome question, that item was not included in the numerator or denominator of the outcome measure index calculated for that participant. Overall, the number of missing responses was very small (0.4% of responses to these questions). Some of the items in the health-related-behavior and financial-outcomes measures were contingent on other questions (e.g., “Have you missed a medication?” is contingent on “Do you take a prescription medication?” and “Do you know your credit card limit?” is contingent on “Do you have a credit card?”). We recoded responses to situational questions depending on their responses to contingent questions. In this case, for example, if participants had a missing or “no” response on “Do you take a prescription medication?” but reported “yes” on “Have you missed a medication?”, we changed the missing or “no” response to “yes”. Our choice then can alter the classification of other responses that were contingent on the same question. For example, if participants did not indicate that they had a credit card, but did know their credit card balance, limit, and/or interest rate, we changed their response to “Do you have a credit card?” to yes. Then, we would include their answers to all credit card questions. In another example, if people did not report whether or not they had sex, but did report that they had had unprotected sex, we changed the response to “Have you had sex in the past 3 months?” to yes, and then included the questions about worrying about STDs and pregnancy. This rule only necessitated changing a small number of missing or “no” responses to “yes” responses (approximately 2.3% of responses to situational questions). We did not change the response to the “had sex in the past 3 months” question for worrying about STDs and pregnancy (which could reasonably occur without having had sex that recently) and having more than $5,000 in credit card debt (which could occur without having a current credit card).

*Financial literacy.* We asked five financial literacy questions (see S3 Table). One financial literacy question (“at the end of the year which person will typically end up with the most money?”) was intended to assess people’s understanding that debt typically carries higher interest rates than savings, so that someone with no debt will be better off than someone with the same net worth but some debt. However, when coding these responses later, we could not ascertain whether “most money” was interpreted by participants to mean total savings, amount earned in interest, or net worth. In addition, although debt generally carries higher interest rates than savings, participants (who are familiar with very low interest student loans) may not have made this assumption. Furthermore, the other four literacy items were correlated with one another (average r=.17), but this item did not (its average correlation with the other items was r=.01). Thus, we removed it from the final index. The final results, however, were similar with or without the item.

Retention analysis

Of the 324 participants enrolled in one of the three statistics classes, 281 consented and completed at least some Time-1 measures; 221 participated in the intervention and are included in the present paper. Of those providing at least some Time-1 data, 83% returned at Time 2. Among these participants, we conducted a logistic regression predicting return to the lab at Time 2 from objective numeracy, subjective numeracy, academic term, instructor, gender, intervention condition, and the interaction of each of these variables with intervention condition. Removing non-significant effects one at a time, only gender (*B=*−1.71, *SE=*0.55, Wald χ²(1)=9.65, *p=.*002, odds ratio=.18) and instructor (*B=*−1.07, *SE=*0.56, Wald χ²(1) =3.64, *p=*.057, odds ratio = .34) remained. Men were less likely to return to the lab than women (81% vs. 95%). Ninety-four percent of students returned for one instructor and 89% returned for the other. No significant effects existed of the intervention condition or any interaction with it on return to the lab.

RMANOVA of ONS

The RMANOVA of the ONS index (Fig 1B) showed a main effect of intervention, *F*_1,183_=7.57, *p=.*007, η²=.040), such that those in the affirmation condition had higher ONS scores. Participants in the values affirmation improved over the semester (*M_ONStime1_*=22.44, *M_ONStime2_*=23.87; *F*_1,183_=6.02, *p=.*015, η²=.032), while participants in the control condition did not (*M_ONStime1_*=21.28, *M_ONStime2_*=21.35, *F*<1). The effect of affirmation was significant at Time 2, *F*_1,183_=8.61, *p=.*004, η²=.045. However, the interaction of affirmation and time was not significant, *F*_1,183_=2.49, *p=.*116, η²=.013. There were no effects of covariates.

Thus, results were substantially similar between this RMANOVA and the GEE reported in the main text, but the interaction of time and condition in this RMANOVA did not reach significance due perhaps to methodological differences between the GEE (which uses all available data and controls for ONS subscales and its interactions) vs. RMANOVA (which deletes cases with missing data and controls for time interactions with the two covariates).

Initial structural equation models

In the main text, we reported a final SEM, after removing nonsignificant effects one at a time. The starting model included a path from the intervention to ONS, SNS, and each of the outcome variables (the latter was intended to examine possible global effects of the intervention, independent of intervention effects mediated by numeracy). Also included were paths from ONS to SNS and to each of the outcome variables, a path from SNS to each of the outcome variables, and paths from our control variables (i.e., ethnicity and Time-1 Financial Outcomes) to ONS, SNS, and each of the outcome variables.

The initial full model for decision-related outcomes was a saturated model, so the degrees of freedom, chi-square, and RMSEA all equaled 0, with AIC=1,996.17 and BIC=2,110.55 (see S1 Fig and S7 Table). The difference in BIC indicated that the full model was an inferior fit to the model reported in text (2,110.55−2,053.50= 57.05).

The full model for STEM-related outcomes was also a saturated model, so that the degrees of freedom, chi-square, and RMSEA all equaled 0, with AIC = 4,106.10 and BIC = 4,223.58 (see S2 Fig and S7 Table). This model included Time-1 financial outcomes as a control variable, but Time-1 financial outcomes did not predict any of the variables in the model and was dropped.

Alternative models: SNS → ONS models

In the main text, we reported the results for ONS → SNS; what follows are the alternative SNS → ONS models. For both decision-related outcomes and STEM-related outcomes, the alternative direction produced similar results (see S3 Fig. and S4 Fig.). Two exceptions existed. In the present models, the intervention produced greater SNS and ONS directly at Time 2 (in the models reported in the main text, the intervention produced only ONS directly), and greater SNS produced greater ONS independent of the intervention’s direct effect on ONS.

The final SEM for decision-related outcomes was a good fit to the data (see Fig. 1; n=194; χ(²(11)=5.08, p=.927; RMSEA= 0.00 [90%CI: 0.00 to 0.02]; CFI = 1.00; AIC= 1,979.26; BIC = 2,057.69), but less well than the ONS → SNS model, as indicated by the larger BIC (2,057.69-2,053.50= 4.19, which is positive evidence that the first model is superior).

The alternative model with affirmation leading to SNS leading to ONS to the STEM outcome variables also fit the data well (n=218; χ²(7)=5.06, p=.652; RMSEA= 0.00 [90%CI: 0.00 to 0.07]; CFI = 1.00; AIC= 4,197.94; BIC = 4,275.79). Strong evidence existed that the ONS → SNS model fit better than the SNS → ONS model; the BIC for the ONS→SNS model was substantially smaller than the BIC for the model reported here (4,275.79– 4,260.72= 15. 07). Because ONS did not predict any of the outcomes, it was also allowed to covary with them. ONS and grades had a significant covariance (see S4 Fig.). ONS did not covary with either intentions or enrollment in additional math classes (*p*s>.32).

Item-by-item analysis of financial-outcome and health-related behavior items

We also conducted a descriptive examination of individual outcome item responses by time and intervention condition (see Table S6). Among intervention participants, the proportion of healthy behaviors increased or stayed the same from Time 1 to Time 2 for eight of the ten health outcomes; two of these outcomes increased by more than 4% (e.g., worry about unplanned pregnancy, frequency of exercise). The remaining two health-related behaviors demonstrated the opposite effect for intervention participants (eating fruits and vegetables and worry about a sexually transmitted disease dropped, respectively, by 7.1% and 13.2% from Time 1 to Time 2). Among control participants, the pattern was quite different; 7 of the 10 health-related behaviors dropped by more than 4%, and only one behavior (forgot medication) increased by more than 4% from Time 1 to Time 2.

For the four financial literacy items, performance improved for intervention participants on two items and declined on two other items; for control participants, performance declined on all four items. Overall, intervention participants did better than controls on every item; they either declined less than controls (2 items) or improved while controls declined (2 items).

For the financial outcome items, half of the items showed an advantage for intervention participants and the other half showed an advantage for control participants. In addition, only 2 out of the 10 financial outcomes showed improvement for intervention participants greater than 4%, and 4 out of the 10 outcomes had declines of more than 4%. Control participants had fewer outcomes improve by more than 4% (only 1 did).

Were control participants less conscientious and put less effort into Time-2 tasks?

The preponderance of protective effects may raise a concern that control participants became less conscientious by the end of the academic term and put less effort into Time-2 tasks than affirmed participants as opposed to the proposed explanation that the intervention protected or improved numeracy. If true, this alternative explanation would predict that control participants would have put less effort into every task (as opposed to only the numeracy-related tasks). If the effects were due instead to values-affirmation in a statistics course as a numeracy intervention, however, we would expect that control participants would put less effort into tasks related to numeracy, but no effect of condition would exist on tasks unrelated to numeracy. Inconsistent with this alternative explanation, no effects existed of condition (or the interaction of time and condition) on science literacy (F<1).

To further examine the alternative explanation, we performed an RMANOVA on time spent on Time-2 tasks, with ethnicity and Time-1 financial outcomes entered as covariates. We followed the recommendations of Ratcliff [1993] and used a cutoff of one standard deviation to remove outliers on time spent on part of the ONS measure (we only measured response time for the part of the ONS measure that involved symbolic arithmetic) and science literacy. This analysis showed a main effect of condition, F_1,168_=4.38, *p*=.04, η² = .025, such that those in the intervention condition took longer than those in the control condition, and a main effect of task, such that the ONS task took longer, F_1,168_=22.85, *p*<.01, η² = .120. However, both effects were qualified by a task × condition interaction, F_1,168_=5.88, *p*=.02, η² = .034; no effect of condition existed on time spent on the science literacy task (F<1), while participants in the values-affirmation condition spent significantly longer on the ONS task than did those in the control condition (M_values-affirmation_ = 254.54 seconds, M_control_ = 216.53 seconds), F_1,168_=5.51, *p=*.02, η² = .032.

Finally, one of the very last questions in the present study asked participants to guess the hypothesis. We counted the number of words participants wrote, with participants who did not write anything receiving a score of 0. The number of words written did not differ by condition (*M*_values-affirmation_ = 12.11 words, *M*_control_ = 13.04 words; F<1).

Gender and sexist stereotypes

Miyake et al. (22) found that values affirmation improved grades in an advanced physics classroom only for female students who held a negative stereotype of women and physics. Based on these and other similar findings, we performed regression analyses on Time-2 objective numeracy, subjective numeracy, and trait math anxiety using gender, values-affirmation condition, Time-1 sexist math stereotypes (mean-centered), and all interactions as predictors (controlling for ethnicity and Time-1 financial outcomes). No statistically significant effects emerged for ONS (ts<1.9), subjective numeracy (ts<2.0) or math anxiety (ts<1). The lack of a stereotype-threat effect is not surprising given the large preponderance of women in this course (75%; 35, 36).
