# Supplementary material for: Improving numeracy through values affirmation enhances decision and STEM outcomes
Source: PLoS One. 2017 Jul 12;12(7):e0180674. doi: 10.1371/journal.pone.0180674 (PMC5507517; doi:10.1371/journal.pone.0180674)
Supplement: S1 Table — Descriptives for all measures discussed in the main text by time. (DOCX) [file pone.0180674.s002.docx]

**Table S1. Descriptive statistics for measures**. Descriptives for all measures discussed in the main text by time. Descriptives are restricted to those who participated in the intervention vs. control manipulation (*n=*221, but *n* below will vary depending on how many participants provided data for each measure). Measures occur in the order that they were discussed in the main text.

| variable |  | N | mean (SD) | range | Cronbach’s α |
| --- | --- | --- | --- | --- | --- |
| Subj numeracy (SNS) | Time 1 | 218 | 4.29 (0.75) | 2.13, 5.75 | .76 |
|  | Time 2 | 201 | 4.22 (0.84) | 1.75, 6 | .81 |
|  |  |  |  |  |  |
| Obj numeracy (ONS) | Time 1 | 210 | 21.78 (4.84) | 7, 31 | .82^^[[1]](#footnote-1)^^ |
|  | Time 2 | 199 | 22.73 (5.74) | 1, 31 | .88^^[[2]](#footnote-2)^^ |
| Final grades in stats course | | 215 | 80.03 (13.21) | 17.40, 97.91 |  |
| Intentions to take additional math classes | | | |  |  |
|  | Time 1 | 211 | 2.33 (1.92) | 0, 6 |  |
|  | Time 2 | 200 | 2.27 (2.06) | 0, 6 |  |
| Math classes per term, before/during stats course | | 194 | 0.52 (0.29) | 0, 1.6 |  |
| Math classes per term, after stats course | | 186 | 0.14 (0.27) | 0, 1.6 |  |
| Health-related behaviors | |  |  |  |  |
|  | Time 1 | 212 | 0.66 (0.18) | 0.2, 1 |  |
|  | Time 2 | 201 | 0.64 (0.18) | 0.17, 1 |  |
| Financial literacy | |  |  |  |  |
|  | Time 1 | 212 | 2.12 (1.05) | 0, 4 |  |
|  | Time 2 | 200 | 1.88 (1.16) | 0, 4 |  |
| Financial outcomes | |  |  |  |  |
|  | Time 1 | 212 | 0.80 (0.19) | 0.20, 1 |  |
|  | Time 2 | 201 | 0.79 (0.21) | 0, 1 |  |
| Working memory | | 211 | 5.17 (1.29) | 2, 9 |  |
| Vocabulary |  | 212 | 19.54 (5.32) | 1, 30 | .82 |
| Science literacy |  |  |  |  |  |
|  | Time 1 | 212 | 6.83 (1.11) | 2, 8 |  |
|  | Time 2 | 200 | 6.68 (1.32) | 2, 8 |  |
| Trait math anxiety | |  |  |  |  |
|  | Time 1 | 218 | 2.51 (0.66) | 1, 4 | .90 |
|  | Time 2 | 201 | 2.50 (0.66) | 1, 4 | .90 |
| Sexist stereotypes | | | | | |
|  | Time 1 | 218 | 2.46 (1.09) | 1,5 |  |
|  | Time 2 | 201 | 2.60 (1.05) | 1, 5 |  |

1. For symbolic arithmetic, Cronbach’s α = .79; for traditional ONS items, Cronbach’s α=.63 [↑](#footnote-ref-1)
2. For symbolic arithmetic, Cronbach’s α = .87; for traditional ONS items, Cronbach’s α=.76 [↑](#footnote-ref-2)
