# Supplementary material for: Improving numeracy through values affirmation enhances decision and STEM outcomes
Source: PLoS One. 2017 Jul 12;12(7):e0180674. doi: 10.1371/journal.pone.0180674 (PMC5507517; doi:10.1371/journal.pone.0180674)
Supplement: S2 Table — Descriptive statistics (frequency and percentage or mean and standard deviation) for all measures and demographics for each condition at each time. (DOCX) [file pone.0180674.s003.docx]

**Table S2. Measures and demographics by time and experimental condition**. For demographic variables, we report frequency (%). For continuous measures at Time 1 and Time 2, we report mean (SD).

| Variable | | Intervention | | Control | | | | Test statistic, p value | | | |
| --- | --- | --- | --- | --- | --- | --- | --- | --- | --- | --- | --- |
| Demographics | | | | | | | | | | |  |
| Gender |  | |  | |  | | | |  | |  |
|  | Men | | 28 (25.5%) | | 25 (23.1%) | | | | χ² (1)= 0.16, *p>.*250 | |  |
|  | Women | | 82 (74.5%) | | 83 (76.9%) | | | |  | |  |
| Race |  | |  | | | |  | | |  |  |
|  | White | | 87 (79.1%) | | | 67 (62.0%) | | | χ² (1) =7.64, *p=.*007 | |  |
|  | Non-white | | 23 (20.9%) | | | 41 (38.0%) | | |  | |  |
| Instructor |  | |  | | |  | | |  | |  |
|  | A | | 47 (43.1%) | | | 45 (42.5%) | | | χ² (1)= 0.01, *p>.*250 | |  |
|  | B | | 62 (56.9%) | | | 61 (57.5%) | | |  | |  |
| Academic term | | |  | | |  | | |  | |  |
|  | Fall | | 79 (70.5%) | | | 76 (69.7%) | | | χ² (1)= 0.02, *p>.*250 | |  |
|  | Winter | | 33 (29.5%) | | | 33 (30.3%) | | |  | |  |
| Time 1 | | | | | | | | | | | |
| SNS | | 4.33 (0.71) | | 4.25 (0.78) | | | | *t*(216)=0.78, *p>.*250 | | | |
| ONS | | 22.27 (4.37) | | 21.28 (5.24) | | | | *t*(208)=1.50, *p=*.136 | | | |
| Math intentions | | 2.31(1.86) | | 2.34 (1.99) | | | | *t*(209)= −0.09, *p>.*250 | | | |
| Math classes per term (before/during) | | 0.52 (0.30) | | 0.53 (0.28) | | | | *t*(192)= −0.25, *p>.*250 | | | |
| Health-related behaviors | | 0.65 (0.19) | | 0.67 (0.18) | | | | t(210)= −0.62, *p>.*250 | | | |
| Financial literacy | | 2.06 (1.18) | | 2.19 (0.90) | | | | *t*(210)= −0.94, *p>.*250 | | | |
| Financial outcomes | | 0.78 (0.19) | | 0.82 (0.18) | | | | *t*(210)= −1.53, *p*=.13 | | | |
| Working memory | | 5.08 (1.27) | | 5.26 (1.31) | | | | *t*(209)=−0.99, *p>.*250 | | | |
| Vocabulary | | 19.68 (4.58) | | 19.40 (6.02) | | | | *t*(210)=0.37, *p>.*250 | | | |
| Science literacy | | 6.80 (1.17) | | 6.87 (1.05) | | | | t(210)= −0.45, *p>.*250 | | | |
| Math anxiety | | 2.48 (0.67) | | 2.54 (0.65) | | | | *t*(216)=−0.63, *p>.*250 | | | |
| Sexist stereotypes | | 2.57 (1.04) | | 2.36 (1.14) | | | | *t*(216) = 1.44, *p>.*250 | | | |
| Time 2 | | | | | | | | | | | |
| SNS | | 4.36 (0.83) | | 4.08 (0.83) | | | | *t*(199)=2.35, *p=.*020 | | | |
| ONS | | 23.85 (4.53) | | 21.51 (6.63) | | | | *t*(197)= 2.93, *p=*0.004 | | | |
| Grades | | 80.03 (13.55) | | 80.03 (12.92) | | | | *t*(213)=0.00, *p>.*250 | | | |
| Math intentions | | 2.36(2.11) | | 2.18 (2.01) | | | | *t*(198)=0.63, *p>.*250 | | | |
| Math classes per term (after) | | 0.11 (0.26) | | 0.16 (0.28) | | | | *t*(184)= −1.15, *p>.*250 | | | |
| Health-related behaviors | | 0.64 (0.19) | | 0.63 (0.19) | | | | *t*(199)= 0.49, *p>.*250 | | | |
| Financial literacy | | 2.01 (1.13) | | 1.74 (1.19) | | | | *t*(198)=1.66, *p=.*098 | | | |
| Financial outcomes | | 0.76 (0.22) | | 0.81 (0.19) | | | | *t*(199)= −1.92, *p=.*056 | | | |
| Science literacy | | 6.61 (1.40) | | 6.76 (1.24) | | | | t(198)= −0.79, *p>.*250 | | | |
| Math anxiety | | 2.48 (0.65) | | 2.53 (0.67) | | | | *t*(199)=−0.61, *p>.*250 | | | |
| Sexist stereotypes | | 2.71 (1.01) | | 2.48 (1.08) | | | | *t*(199)= 1.59, *p=.*112 | | | |
