# Supplementary material for: Improving numeracy through values affirmation enhances decision and STEM outcomes
Source: PLoS One. 2017 Jul 12;12(7):e0180674. doi: 10.1371/journal.pone.0180674 (PMC5507517; doi:10.1371/journal.pone.0180674)
Supplement: S3 Table — (DOCX) [file pone.0180674.s004.docx]

**Table S3. ONS items with scoring criteria and percentage correct**. Some items have multiple correct responses, including ranges.

| subscale | item wording | correct response | Time 1 | Time 2 |
| --- | --- | --- | --- | --- |
| traditional ONS | Imagine that we roll a fair, six-sided die 1,000 times. Out of 1,000 rolls, how many times do you think the die would come up as an even number? | 490-510 | 87.2% | 85.0% |
| traditional ONS | In the BIG BUCKS LOTTERY, the chances of winning a $10.00 prize are 1%. What is your best guess about how many people would win a $10.00 prize if 1,000 people each buy a single ticket from BIG BUCKS? | 10 | 80.6% | 79.5% |
| traditional ONS | Which of the following numbers represents the biggest risk of getting a disease? ___ 1 in 100 ___ 1 in 1000 ___ 1 in 10 | 1 in 10 | 95.3% | 96.5% |
| traditional ONS | Which of the following numbers represents the biggest risk of getting a disease? ___ 1% __ 10% ___ 5% | 10% | 99.5% | 95.5% |
| traditional ONS | If Person A’s risk of getting a disease is 1% in ten years, and Person B’s risk is double that of A’s, what is B’s risk? ___% in _____ years | 2 in 10, 1 in 5, 4 in 20 | 81.5% | 76.0% |
| traditional ONS | If the chance of getting a disease is 10%, how many people would be expected to get the disease: A: Out of 100? | 10 | 93.8% | 92.0% |
| traditional ONS | If the chance of getting a disease is 10%, how many people would be expected to get the disease: B: Out of 1000? | 100 | 83.4% | 81.0% |
| traditional ONS | If the chance of getting a disease is 20 out of 100, this would be the same as having a __% chance of getting the disease. | 20 | 89.6% | 91.5% |
| traditional ONS | The chance of getting a viral infection is .0005. Out of 10,000 people, about how many of them are expected to get infected? | 5 | 52.6% | 64.0% |
| traditional ONS | Which of the following numbers represents the biggest risk of getting a disease? __ 1 chance in 12 ___ 1 chance in 37 | 1 in 12 | 96.7% | 95.0% |
| traditional ONS | mammogram question (see below) | see below | 27.0% | 22.5% |
| traditional ONS | Imagine that you are taking a class and your chances of being asked a question in class are 1% during the first week of class and double each week thereafter (i.e., you would have a 2% chance in Week 2, a 4% chance in Week 3, an 8% chance in Week 4). What is the probability that you will be asked a question in class during Week 7? ___% | 64 | 77.7% | 71.5% |
| traditional ONS | SARS question (see below) | see below | 61.6% | 53.0% |
| traditional ONS | A bat and a ball cost $1.10 in total. The bat costs $1.00 more than the ball. How much does the ball cost? ________ cents | 5 | 32.2% | 37.5% |
| traditional ONS | If it takes 5 machines 5 minutes to make 5 widgets, how long would it take 100 machines to make 100 widgets? _______widgets | 5 | 16.1% | 22.5% |
| traditional ONS | In a lake, there is a patch of lilypads. Every day, the patch doubles in size. If it takes 48 days for the patch to cover the entire lake, how long would it take for the patch to cover half of the lake? _____ days | 47 | 30.3% | 31.5% |

| subscale | item wording | correct response | Time 1 | Time 2 |
| --- | --- | --- | --- | --- |
| sym. arith. | 48 – 19 = | 29 | 88.9% | 91.0% |
| sym. arith. | 14 × 6 = | 84 | 85.3% | 86.4% |
| sym. arith. | 2/3 – 1/3 = | 1/3 or (.33, .333, .3333,…, .333333) | 97.2% | 95.5% |
| sym. arith. | 126 ÷ 42 = | 3 | 78.3% | 81.9% |
| sym. arith. | 288 ÷ 48 = | 6 | 57.1% | 76.9% |
| sym. arith. | 7/8 – 2/8 = | 5/8 or .625 | 98.2% | 94.0% |
| sym. arith. | 3250 ÷ 25 = | 130 | 49.3% | 72.9% |
| sym. arith. | 2 ¾ + 4 1/8 = | 7/8 or 6.875 | 61.3% | 54.3% |
| sym. arith. | 1.08 × .2 = | .216 | 30.4% | 55.8% |
| sym. arith. | 2 6/7 – 5 ½ = | −37/14 or (−2.6, −2.64, −2.643, …,−2.642857143) | 18.9% | 22.6% |
| sym. arith. | -18 + 12 = | 6 | 93.6% | 86.4% |
| sym. arith. | -6 × 7 = | −42 | 93.1% | 86.9% |
| sym. arith. | 4/7 ÷ ½ = | 8/7 or (1.1, 1.14, 1.143,…, 1.142857143) | 41.0% | 53.3% |
| sym. arith. | The average (or mean) of 22 and 9 = | 15.5 | 77.0% | 85.4% |
| sym. arith. | The average (or mean) of 15, 4, and 3 = | 7 1/3 or (7.3, 7.33, 7.333,…,7.33333) | 53.9% | 68.3% |
| sym. arith. | The average (or mean) of 6, 4, 9, and 11 = | 7.5 | 51.6% | 64.8% |

Mammogram question:

Suppose you have a close friend who has a lump in her breast and must have a mammogram. Of 100 women like her, 10 of them actually have a malignant tumor and 90 of them do not. Of the 10 women who actually have a tumor, the mammogram indicates correctly that 9 of them have a tumor and indicates incorrectly that 1 of them does not have a tumor. Of the 90 women who do not have a tumor, the mammogram indicates correctly that 81 of them do not have a tumor and indicates incorrectly that 9 of them do have a tumor. The table below summarizes all of this information. Imagine that your friend tests positive (as if she had a tumor), what is the likelihood that she actually has a tumor?

|  | tested positive | tested negative | totals |
| --- | --- | --- | --- |
| actually has tumor | 9 | 1 | 10 |
| does not have tumor | 9 | 81 | 90 |
| total | 18 | 82 | 100 |

Answer: _____ out of ________ (9 out of 18)

SARS question:

Suppose that 1 out of every 10,000 doctors in a certain region is infected with the SARS virus; in the same region, 20 out of every 100 people in a particular at-risk population also are infected with the virus. A test for the virus gives a positive result in 99% of those who are infected and in 1% of those who are not infected. A randomly selected doctor and a randomly selected person in the at-risk population in this region both test positive for the disease. Who is more likely to actually have the disease?

___ They both tested positive for SARS and therefore are equally likely to have the disease

___ They both tested positive for SARS, and the doctor is more likely to have the disease

_X_ They both tested positive for SARS and the person in the at-risk population is more likely to have the disease.
