# Supplementary material for: Improving numeracy through values affirmation enhances decision and STEM outcomes
Source: PLoS One. 2017 Jul 12;12(7):e0180674. doi: 10.1371/journal.pone.0180674 (PMC5507517; doi:10.1371/journal.pone.0180674)
Supplement: S4 Table — Percentages of positive responses to health-related behaviors, financial outcomes and financial literacy measures. (DOCX) [file pone.0180674.s005.docx]

**Table S4. Health-related behavior, financial outcome and financial literacy measures**. Percentages of responses to health-related behaviors, financial outcomes, and financial literacy measures. These percentages are restricted to the participants who did the intervention (*n*_T1_=212, *n*_T2_=201). Situational questions are numbered with S before the number, and were not included in the index. For questions that were not contingent on another question, a percentage with the positive outcome was calculated. Indented questions are contingent on the question they follow, and the percentage who have positive outcomes was calculated based only on the participants who had the opportunity to experience that outcome. Only questions with positive outcomes (i.e., not questions that indicated opportunity to experience an outcome) were included in analysis. For financial literacy questions, the questions and answer choices are both given, with the correct answer bolded for each question. The percentages indicate correct answers. Item 3 of the financial literacy items was not included in the reported analyses, but was included here for completeness (see S1 supporting information).

| Question | opportunity to experience | | positive outcome | |
| --- | --- | --- | --- | --- |
|  | T1 | T2 | T1 | T2 |
| HEALTH-RELATED BEHAVIORS |  |  |  |  |
| 1. Did you get a flu vaccine this past year? |  |  | 32.1% | 28.4% |
| 1. Do you plan to get a flu vaccine this coming year? |  |  | 37.7% | 36.3% |
| S1. In the past 3 months, have you taken any prescription medications? | 67.0% | 71.1% |  |  |
| 1. In the past 3 months, have you forgotten to take your medication? (% no is indicated) |  |  | 31.0% | 35.7% |
| 1. Do you smoke cigarettes? |  |  | 90.1% | 91.5% |
| S2. Have you had sex in the past 3 months? | 61.8% | 61.2% |  |  |
| 1. In the past 3 months, have you had sex in a non−committed relationship without using a condom? (%no is indicated) |  |  | 78.6% | 75.6% |
| 1. In the past 3 months, have you or your partner worried about an unplanned pregnancy? (%no is indicated) |  |  | 70.2% | 67.7% |
| 1. In the past 3 months, have worried about having a sexually transmitted disease? (%no is indicated) |  |  | 87.2% | 80.2% |
| S3. In the past year, have you been tested for a sexually transmitted disease? | 27.4% | 30.3% |  |  |
| 1. In the past year, have you been diagnosed with a sexually transmitted disease? (%no is indicated) |  |  | 98.3% | 93.4% |
| 1. How often do you work out or exercise each week? (no exercise = 0, one or more times exercising per week = 1) |  |  | 81.6% | 82.1% |
| 1. How many servings of fruits and vegetables do you eat each week? (0-2 servings of fruits and vegetables = 0, 3 or more servings = 1) |  |  | 84.0% | 77.1% |

| Question | opportunity to experience | | positive outcome | |
| --- | --- | --- | --- | --- |
|  | T1 | T2 | T1 | T2 |

| FINANCIAL OUTCOMES |  |  |  |  |
| --- | --- | --- | --- | --- |
| 1. Do you have a savings account or emergency fund? |  |  | 87.3% | 86.6% |
| 1. Do you know how much money you have in your savings or emergency fund? |  |  | 90.8% | 86.8% |
| S1. Do you have a credit card? | 60.4% | 61.7% |  |  |
| 1. Do you know your credit card balance? |  |  | 85.2% | 84.7% |
| 1. Do you know your credit card limit? |  |  | 89.1% | 85.5% |
| 1. Do you know your credit card interest rate? |  |  | 44.5% | 50.0% |
| 1. In the past year, have you had more than $5,000 in credit card debt? (%no is indicated) |  |  | 96.2% | 91.2% |
| 1. In the past year, have you had an overdraft or bounced a check from your account? (%no is indicated) |  |  | 73.1% | 74.6% |
| 1. In the past year, have you used up your funds (e.g., checking, food points) early (for example, before your next paycheck or regular allowance from family)? (%no is indicated) |  |  | 70.3% | 66.7% |
| S2. In the past year, have you been responsible for paying utilities (e.g., electricity, gas, cable, or water)? | 65.6% | 67.7% |  |  |
| 1. In the past year, have you ever had a utility (e.g., electricity, gas, cable, or water) shut off due to late or no payment? (%no is indicated) |  |  | 90.6% | 89.7% |
| S3. Have you ever been responsible for rent or mortgage payments? | 61.3% | 63.7% |  |  |
| 1. Have you ever been late on a rent or mortgage payment? (%no is indicated) |  |  | 80.0% | 75.0% |

|  |  |  |  |
| --- | --- | --- | --- |

| Question |  | |  |
| --- | --- | --- | --- |
|  |  | correctly answered | |

| FINANCIAL LITERACY (correct answers in bold) |  | T1 T2 | | |
| --- | --- | --- | --- | --- |
| 1. Imagine that the interest rate on your savings account was 1% per year and inflation was 2% per year. After 1 year, would you be able to buy more than, exactly the same as, or less than today with the money in this account?    1. more than;    2. same ;    3. **less than** | |  | 65.6% | 56.0% |
| 1. If you invest $1,000 today at 4% for a year, your balance in a year will be:    1. **higher if the interest is compounded daily rather than monthly;**    2. higher if the interest is compounded quarterly rather than weekly;    3. higher if the interest is compounded yearly rather than quarterly;    4. $1,040 no matter how the interest is computed;    5. $1,000 no matter how the interest is computed. | |  | 53.8% | 52.0% |
|  | |  |  |  |
| 1. Considering a long time period (for example 10 or 20 years), which asset normally gives the highest return? 2. Savings accounts; 3. Bonds; 4. **Stocks;** 5. Don’t know; | |  | 19.8% | 17.5% |
| 1. Normally, which asset displays the highest fluctuations over time?    1. Savings accounts;    2. Bonds;    3. **Stocks;**    4. Don’t know | |  | 73.1% | 62.5% |
| 1. (Removed) Using only the numbers below, at the end of the year which person will typically end up with the most money?    1. An individual with $20,000 in debt and $10,000 in savings;    2. An individual with $10,000 in debt and $20,000 in savings;    3. **An individual with no debt and $10,000 in savings;**    4. An individual with $100,000 in debt and $110,000 in savings;    5. b, c, and d will all end up with the same amount of money. | |  | 29.3% | 26.0% |
