# Supplementary material for: Improving numeracy through values affirmation enhances decision and STEM outcomes
Source: PLoS One. 2017 Jul 12;12(7):e0180674. doi: 10.1371/journal.pone.0180674 (PMC5507517; doi:10.1371/journal.pone.0180674)
Supplement: S5 Table — Correlation table of all variables included in analyses, restricted to those who participated in the intervention manipulation. (DOCX) [file pone.0180674.s006.docx]

**Table S5. Correlation table.** Correlation table of all variables included in analyses, restricted to those who participated in the intervention manipulation (*n=*167-221 depending on participants with available data for each measure). **p<*0.05

|  | 1 | 2 | 3 | 4 | 5 | 6 | 7 | 8 | 9 | 10 | 11 | 12 | 13 | 14 | 15 | 16 | 17 | 18 | 19 | 20 | 21 | 22 |
| --- | --- | --- | --- | --- | --- | --- | --- | --- | --- | --- | --- | --- | --- | --- | --- | --- | --- | --- | --- | --- | --- | --- |
| 1. Intervention condition |  |  |  |  |  |  |  |  |  |  |  |  |  |  |  |  |  |  |  |  |  |  |
| 2. ethnicity | 0.19* |  |  |  |  |  |  |  |  |  |  |  |  |  |  |  |  |  |  |  |  |  |
| 3. T1 SNS | 0.05 | 0.01 |  |  |  |  |  |  |  |  |  |  |  |  |  |  |  |  |  |  |  |  |
| 4. T2 SNS | 0.16* | −0.06 | 0.71* |  |  |  |  |  |  |  |  |  |  |  |  |  |  |  |  |  |  |  |
| 5. T1 ONS | 0.10 | −0.03 | 0.50* | 0.53* |  |  |  |  |  |  |  |  |  |  |  |  |  |  |  |  |  |  |
| 6. T2 ONS | 0.20* | 0.01 | 0.26* | 0.36* | 0.45* |  |  |  |  |  |  |  |  |  |  |  |  |  |  |  |  |  |
| 7. Grades | 0.00 | 0.01 | 0.09 | 0.24* | 0.20* | 0.21* |  |  |  |  |  |  |  |  |  |  |  |  |  |  |  |  |
| 8. T1 intent. | −0.01 | −0.01 | 0.42* | 0.46* | 0.38* | 0.18* | 0.11 |  |  |  |  |  |  |  |  |  |  |  |  |  |  |  |
| 9. T2 intent. | 0.04 | −0.07 | 0.41* | 0.50* | 0.35* | 0.23* | 0.22* | 0.71* |  |  |  |  |  |  |  |  |  |  |  |  |  |  |
| 10. Prior math classes | −0.02 | −0.11 | 0.02 | 0.06 | −0.04 | 0.04 | −0.06 | 0.22* | 0.19* |  |  |  |  |  |  |  |  |  |  |  |  |  |
| 11. Future math classes | −0.08 | −0.18* | 0.19* | 0.35* | 0.17* | 0.13 | 0.21* | 0.40* | 0.52* | 0.31* |  |  |  |  |  |  |  |  |  |  |  |  |
| 12. T1 health-related behaviors | −0.04 | 0.06 | 0.06 | 0.08 | 0.08 | 0.05 | 0.05 | 0.06 | 0.03 | 0.02 | −0.04 |  |  |  |  |  |  |  |  |  |  |  |
| 13. T2 health-related behaviors | 0.04 | 0.09 | 0.11 | 0.12 | 0.10 | 0.18* | 0.16* | 0.07 | 0.08 | 0.05 | 0.04 | 0.63* |  |  |  |  |  |  |  |  |  |  |
| 14. T1 fin. lit. | −0.06 | 0.09 | 0.20* | 0.24* | 0.21* | 0.12 | 0.00 | 0.25* | 0.24* | 0.06 | 0.05 | 0.03 | 0.03 |  |  |  |  |  |  |  |  |  |
| 15. T2 fin. lit. | 0.12 | 0.07 | 0.22* | 0.27* | 0.27* | 0.35* | 0.12 | 0.28* | 0.28* | 0.02 | 0.20* | 0.07 | 0.12 | 0.47* |  |  |  |  |  |  |  |  |
| 16. T1 fin. outcomes | −0.11 | 0.05 | 0.05 | −0.01 | 0.07 | 0.03 | 0.09 | 0.01 | 0.00 | −0.04 | 0.04 | 0.09 | 0.15* | 0.10 | 0.05 |  |  |  |  |  |  |  |
| 17. T2 fin. outcomes | −0.14 | 0.16* | 0.02 | 0.07 | 0.07 | 0.18* | 0.16* | 0.09 | 0.10 | 0.05 | 0.02 | 0.11 | 0.18* | 0.12 | 0.11 | 0.65* |  |  |  |  |  |  |
| 18. Work. memory | −0.07 | 0.03 | 0.18* | 0.09 | 0.18* | 0.09 | −0.03 | 0.00 | −0.10 | −0.08 | 0.01 | 0.07 | −0.01 | 0.05 | 0.03 | −0.08 | −0.05 |  |  |  |  |  |
| 19. vocab. | 0.03 | 0.17* | −0.01 | 0.03 | 0.06 | −0.01 | 0.12 | 0.06 | 0.06 | −0.22* | 0.02 | −0.04 | −0.07 | 0.10 | 0.16* | −0.03 | 0.03 | 0.08 |  |  |  |  |
| 20. T1 anxiety | −0.04 | −0.01 | −0.47* | −0.46* | −0.45* | −0.14 | −0.22* | −0.53* | −0.44* | −0.02 | −0.23* | −0.04 | −0.05 | −0.08 | −0.21* | −0.01 | −0.05 | −0.05 | −0.03 |  |  |  |
| 21. T2 anxiety | −0.04 | 0.00 | −0.42* | −0.47* | −0.39* | −0.26* | −0.34* | −0.47* | −0.53* | 0.02 | −0.31* | 0.02 | −0.07 | −0.10 | −0.22* | 0.01 | −0.10 | −0.02 | −0.02 | 0.78* |  |  |
| 22. T1 sexism | 0.10 | −0.04 | −0.17* | −0.10 | −0.04 | −0.10 | −0.06 | −0.15* | −0.16* | −0.07 | −0.07 | −0.06 | −0.04 | −0.11 | −0.13 | 0.06 | 0.03 | 0.08 | −0.08 | 0.14* | 0.16* |  |
| 23. T2 sexism | 0.11 | 0.00 | −0.16* | −0.08 | −0.05 | −0.16* | −0.06 | −0.10 | −0.11 | −0.01 | −0.17* | −0.12 | −0.19* | −0.03 | −0.09 | 0.01 | −0.05 | 0.06 | −0.02 | 0.18* | 0.24* | 0.61* |
