# Supplementary material for: Improving numeracy through values affirmation enhances decision and STEM outcomes
Source: PLoS One. 2017 Jul 12;12(7):e0180674. doi: 10.1371/journal.pone.0180674 (PMC5507517; doi:10.1371/journal.pone.0180674)
Supplement: S6 Table — Percentages of responses to health-related behavior, financial outcome, and financial literacy measures by condition and time. (DOCX) [file pone.0180674.s007.docx]

**Table S6. Health-related behavior, financial outcome, and financial literacy measures by condition and time**. Percentages of responses to health-related behavior, financial outcome, and financial literacy measures by condition and time. These percentages are restricted to the participants who did the intervention (*n*_T1_=212, *n*_T2_=201). The questions are ordered from the largest to smallest advantage for the values-affirmation condition. The number shown immediately prior to each question indicates the order in which it was shown to participants.

| Question | Values-affirmation | | Control | | Advantage for affirmed |
| --- | --- | --- | --- | --- | --- |
|  | T1 | T2 | T1 | T2 |  |
| HEALTH-RELATED BEHAVIORS |  |  |  |  |  |
| 1. 6 In the past 3 months, have you or your partner worried about an unplanned pregnancy (no = positive) % no is indicated | 65.2% | 71.0% | 75.4% | 64.5% | +16.7% |
| 1. 9 How often do you work out or exercise each week (no exercise = 0, one or more times exercising per week = 1) | 81.5% | 86.7% | 81.7% | 77.1% | +9.8% |
| 1. 1 Did you get a flu vaccine this past year (yes=positive outcome) %yes is indicated | 32.4% | 32.4% | 31.7% | 24.0% | +7.7% |
| 1. 2 Do you plan to get a flu vaccine this coming year (yes= positive) % yes is indicated | 38.9% | 40.0% | 36.5% | 32.3% | +5.3% |
| 1. 8 In the past year, have you been diagnosed with a sexually transmitted disease (no = positive) % no is indicated | 95.7% | 93.3% | 100% | 93.5% | +4.1% |
| 1. 5 In the past 3 months, have you had sex in a non−committed relationship without using a condom (no = positive) % no is indicated | 72.7% | 71.0% | 84.6% | 80.3% | +2.6% |
| 1. 4 Do you smoke cigarettes (no = positive) % no is indicated | 88.9% | 90.5% | 91.3% | 92.7% | +0.2% |
| 1. 10 How many servings of fruits and vegetables do you eat each week (0-2 servings of fruits and vegetables = 0, 3 or more servings = 1) | 85.2% | 78.1% | 82.7% | 76.0% | -0.4% |
| 1. 3 In the past 3 months, have you forgotten to take your medication (no= positive) % no is indicated | 29.8% | 29.0% | 32.9% | 41.9% | -9.8% |
| 1. 7 In the past 3 months, have worried about having a sexually transmitted disease (no = positive) % no is indicated | 91.0% | 77.8% | 83.3% | 82.5% | -12.4% |
| **# of outcomes that increase by >4% from T1 to T2** |  | **2** |  | **1** |  |
| **# of outcomes that decrease by >4% from T1 to T2** |  | **2** |  | **7** |  |

| Question | Values-affirmation | | Control | | Advantage for affirmed |
| --- | --- | --- | --- | --- | --- |
|  | T1 | T2 | T1 | T2 |  |
| FINANCIAL LITERACY (correct answers in bold, item 3 removed, see S1 supporting information) |  |  |  | |  |
| 1. 2 If you invest $1,000 today at 4% for a year, your balance in a year will be: 2. **higher if the interest is compounded daily rather than monthly;** 3. higher if the interest is compounded quarterly rather than weekly; 4. higher if the interest is compounded yearly rather than quarterly; 5. $1,040 no matter how the interest is computed; 6. $1,000 no matter how the interest is computed. | 50.0% | 56.2% | 57.7% | 46.9% | +17.0% |
| 1. 5 Normally, which asset displays the highest fluctuations over time? 2. Savings accounts; 3. Bonds; 4. **Stocks;** 5. Don’t know; | 74.1% | 67.6% | 72.1% | 56.3% | +9.3% |
| 1. 1 Imagine that the interest rate on your savings account was 1% per year and inflation was 2% per year. After 1 year, would you be able to buy more than, exactly the same as, or less than today with the money in this account? 2. more than; 3. same ; 4. **less than** | 63.9% | 58.1% | 67.3% | 53.1% | +8.4% |
| 1. 4 Considering a long time period (for example 10 or 20 years), which asset normally gives the highest return? 2. Savings accounts; 3. Bonds; 4. **Stocks;** 5. Don’t know; | 17.6% | 19.0% | 22.1% | 15.6% | +7.9% |
| **# of outcomes that increase by >4% from T1 to T2** |  | **1** |  | **0** |  |
| **# of outcomes that decrease by >4% from T1 to T2** |  | **2** |  | **4** |  |

| Question | | Values-affirmation | | | | Control | | | | Advantage for affirmed |
| --- | --- | --- | --- | --- | --- | --- | --- | --- | --- | --- |
|  | | T1 | | T2 | | T1 | | T2 | |  |
| FINANCIAL OUTCOMES |  | |  | | |  |  | | |  |
| 1. 3 Do you know your credit card balance (yes=positive outcome) %yes is indicated | 85.3% | | 88.1% | | | 85.0% | 80.7% | | | +7.1% |
| 1. 7 In the past year, have you had an overdraft or bounced a check from your account (no=positive outcome) % no is indicated | 72.2% | | 77.1% | | | 74.0% | 71.9% | | | +7.0% |
| 1. 10 Have you ever been late on a rent or mortgage payment (no=positive outcome) % no is indicated | 78.1% | | 74.2% | | | 81.8% | 75.8% | | | +2.1% |
| 1. 6 In the past year, have you had more than $5,000 in credit card debt (no=positive outcome) % no is indicated | 97.1% | | 92.6% | | | 95.1% | 89.5% | | | +1.1% |
| 1. 4 Do you know your credit card limit (yes=positive outcome) %yes is indicated | 86.8% | | 83.6% | | | 91.7% | 87.7% | | | +0.8% |
| 1. 2 Do you know how much money you have in your savings or emergency fund (yes=positive outcome) %yes is indicated | 88.3% | | 83.9% | | | 93.4% | 89.7% | | | -0.7% |
| 1. 5 Do you know your credit card interest rate (yes=positive outcome) %yes is indicated | 45.6% | | 50.7% | | | 43.3% | 50.9% | | | -2.5% |
| 1. 9 In the past year, have you ever had a utility (e.g., electricity, gas, cable, or water) shut off due to late or no payment (no=positive outcome) % no is indicated | 88.7% | | 86.1% | | | 92.6% | 93.8% | | | -3.8% |
| 1. 1 Do you have a savings account or emergency fund (yes=positive outcome) %yes is indicated | 87.0% | | 82.9% | | | 87.5% | 90.6% | | | -7.2% |
| 1. 8 In the past year, have you used up your funds (e.g., checking, food points) early (for example, before your next paycheck or regular allowance from family) (no=positive outcome) % no is indicated | 70.4% | | 62.9% | | | 70.2% | 70.8% | | | -8.1% |
| **# of outcomes that increase by >4% from T1 to T2** | |  | | **2** |  | | | **1** |  | |
| **# of outcomes that decrease by >4% from T1 to T2** | |  | | **4** |  | | | **4** |  | |
