# Supplementary material for: Improving numeracy through values affirmation enhances decision and STEM outcomes
Source: PLoS One. 2017 Jul 12;12(7):e0180674. doi: 10.1371/journal.pone.0180674 (PMC5507517; doi:10.1371/journal.pone.0180674)
Supplement: S7 Table — Beta coefficients (standard error in parentheses) of the effects of covariates (i.e., ethnicity and Time-1 Financial Outcomes) on variables in full SEM model (see S1 Supporting Information, S1 and S3 Figs). (DOCX) [file pone.0180674.s008.docx]

**Table S7. Beta coefficients for starting SEM models**. Beta coefficients (standard error in parentheses) of the effects of covariates (i.e., ethnicity and Time-1 Financial Outcomes) on variables in full SEM model (see S1 Supporting information, S1 Fig, and S3 Fig). *p<.05, †p<.10. ONS= objective numeracy; SNS = subjective numeracy.

|  | ethnicity | T1 Financial outcomes |
| --- | --- | --- |
| Decision outcomes |  |  |
| ONS | −.22 (.92) | 1.97 (2.36) |
| SNS | −.10 (.12) | −.02 (.34) |
| health behaviors | .03 (.03) | .14 (.08) † |
| financial outcomes | .07 (.03)* | .71 (.07)* |
| financial literacy | .17 (.16) | .22 (.46) |
|  |  |  |
| STEM outcomes |  |  |
| ONS | −.38 (.95) | 2.30 (2.40) |
| SNS | −.11 (.12) | .02 (.33) |
| additional classes | −.10 (.05) † | .01 (.09) |
| intentions | −.19 (.30) | .04 (.77) |
| grades | .34 (1.77) | 4.86 (6.21) |
