# Supplementary figures and images for: Improving numeracy through values affirmation enhances decision and STEM outcomes
Source: PLoS One. 2017 Jul 12;12(7):e0180674. doi: 10.1371/journal.pone.0180674 (PMC5507517; doi:10.1371/journal.pone.0180674)

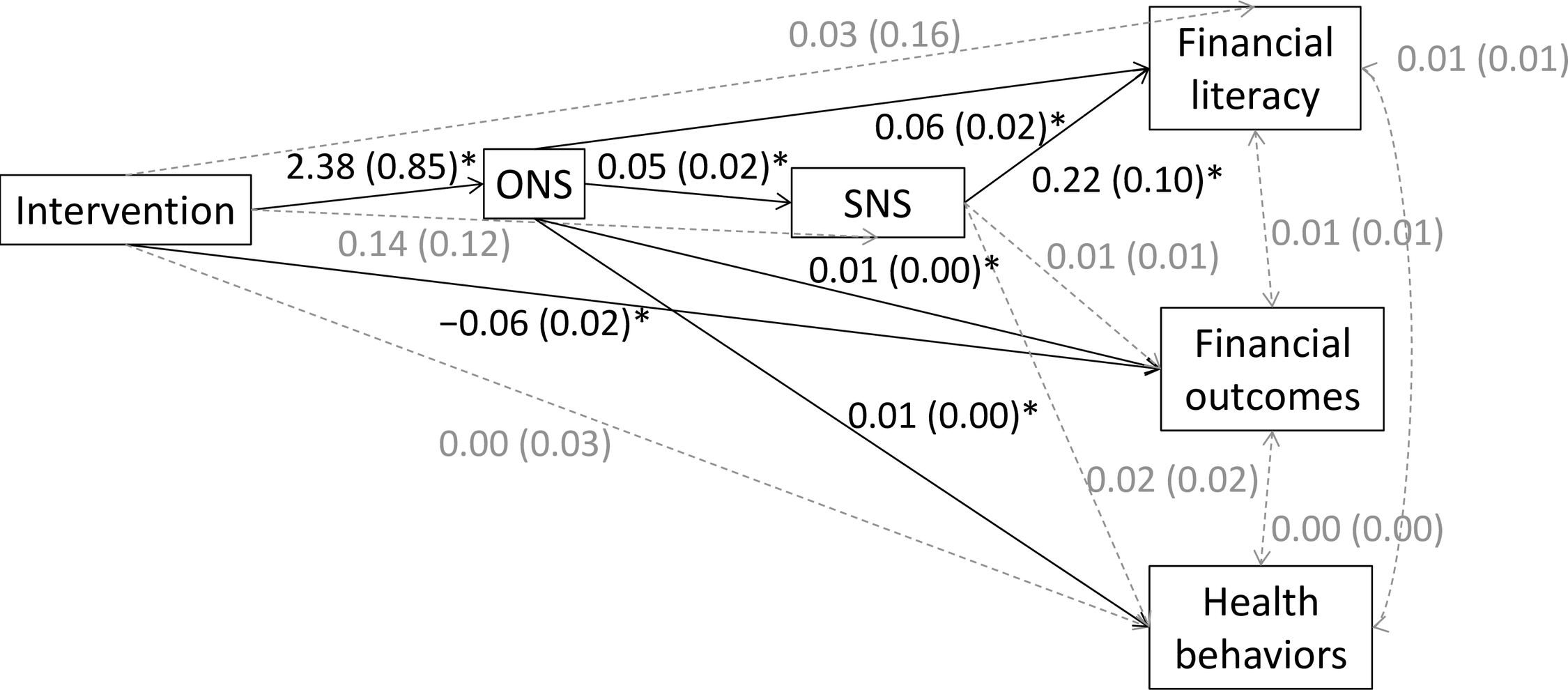

Supplement: S1 Fig — Structural equation model of the effects of the numeracy intervention on all Time-2 decision outcomes (i.e., financial literacy, health-related behaviors, financial outcomes, n = 194). Path coefficients are unstandardized. Solid paths are p < .0.10, *p < .05, †p < .10, dashed paths are p>.10. Paths from Time-1 financial outcomes and ethnicity are not shown in figure and can be found in S7 Table. ONS = objective numeracy; SNS = subjective numeracy. (TIF) [file pone.0180674.s009.tif]

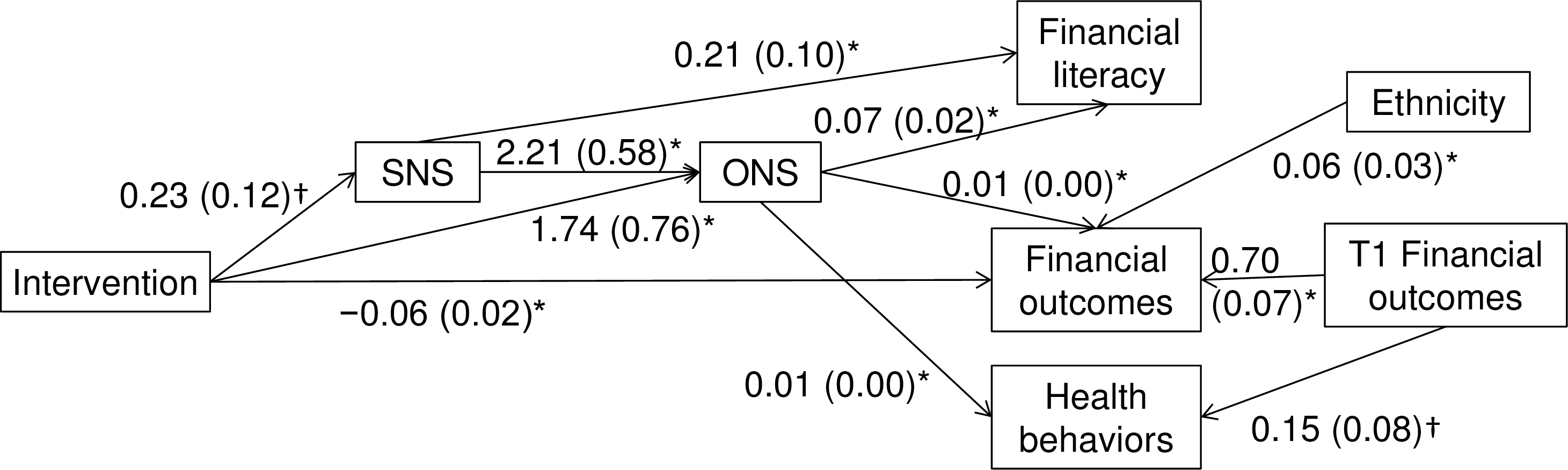

Supplement: S2 Fig — Structural equation model of the effects of the numeracy intervention on all Time-2 decision outcomes (i.e., financial literacy, health-related behaviors, financial outcomes, n = 194). Path coefficients are unstandardized; standard errors are in parentheses. Paths are *p < .050, †p < .010. Nonsignificant paths (p>.100) were removed from the final model and are not shown. ONS = objective numeracy; SNS = subjective numeracy. (TIF) [file pone.0180674.s010.tif]

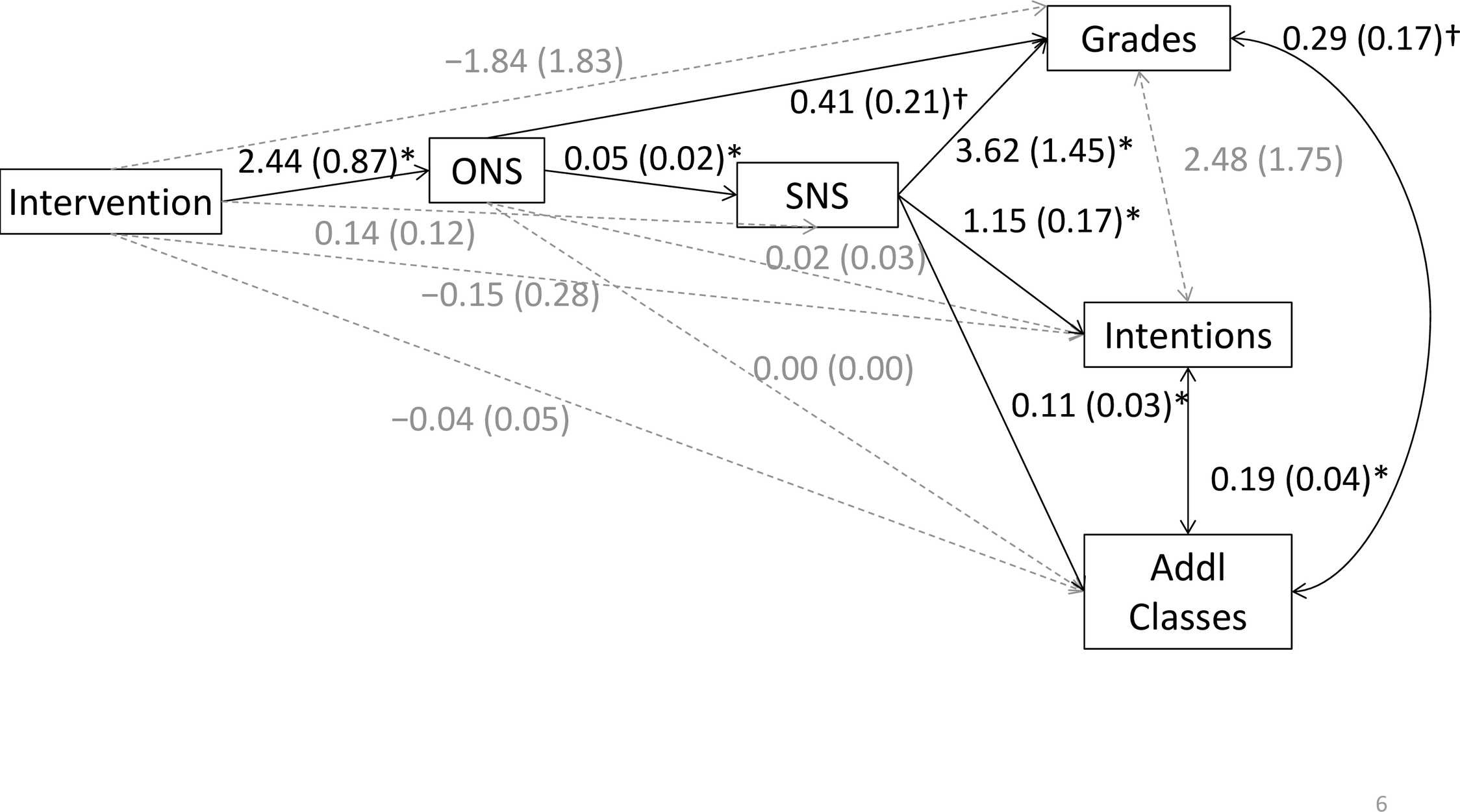

Supplement: S3 Fig — Structural equation model of the effects of the numeracy intervention on all Time-2 STEM-related outcomes (i.e., grades in the statistics course, Time-2 intentions to take more math classes, and number of math-intensive courses per term after the experiment, n = 212). Path coefficients are unstandardized; standard errors are in parentheses. Solid paths are *p < .05, †p < .10, dashed paths are p>.10. Paths from Time-1 financial outcomes and ethnicity are not shown in figure and can be found in S7 Table. ONS = objective numeracy; SNS = subjective numeracy. (TIF) [file pone.0180674.s011.tif]

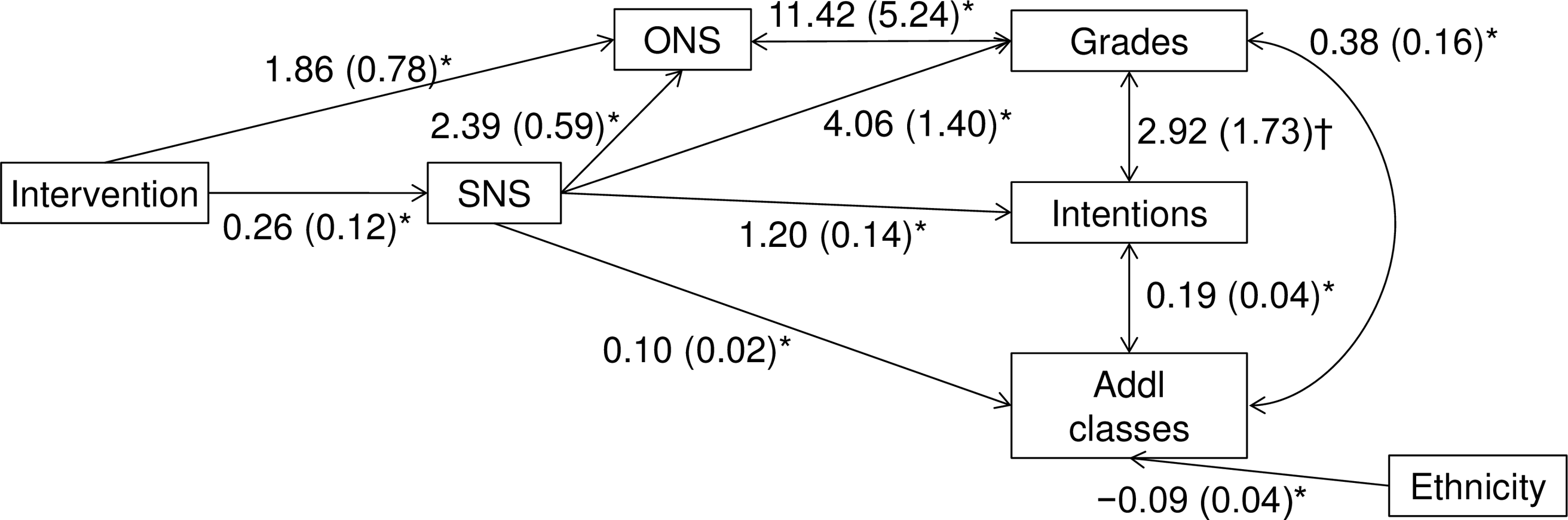

Supplement: S4 Fig — Structural equation model of the effects of the numeracy intervention on all Time-2 STEM-related outcomes (i.e., grades in the statistics course, Time-2 intentions to take more math classes, and number of math-intensive courses per term after the experiment, n = 218). Path coefficients are unstandardized; standard errors are in parentheses. Paths are *p < .050, †p < .010. Nonsignificant paths (p>.100) were removed from the final model and are not shown. ONS = objective numeracy; SNS = subjective numeracy. (TIF) [file pone.0180674.s012.tif]
